# Polymer Coated Oncolytic Adenovirus to Selectively Target Hepatocellular Carcinoma Cells

**DOI:** 10.3390/pharmaceutics13070949

**Published:** 2021-06-24

**Authors:** Mariangela Garofalo, Federica Bellato, Salvatore Magliocca, Alessio Malfanti, Lukasz Kuryk, Beate Rinner, Samuele Negro, Stefano Salmaso, Paolo Caliceti, Francesca Mastrotto

**Affiliations:** 1 Department of Pharmaceutical and Pharmacological Sciences, University of Padova, Via F. Marzolo 5, 35131 Padova, Italy; federica.bellato@unipd.it (F.B.); magliocca.s@gmail.com (S.M.); stefano.salmaso@unipd.it (S.S.); paolo.caliceti@unipd.it (P.C.); 2 Université Catholique de Louvain, Louvain Drug Research Institute, Advanced Drug Delivery and Biomaterials, Avenue Mounier, 73 bte B1 73.12, 1200 Brussels, Belgium; alessio.malfanti@uclouvain.be; 3 Department of Virology, National Institute of Public Health—National Institute of Hygiene, Chocimska 24, 00-791 Warsaw, Poland; lkuryk@pzh.gov.pl; 4 Clinical Science, Targovax Oy, Saukonpaadenranta 2, 00180 Helsinki, Finland; 5 Division of Biomedical Research, Medical University of Graz, Roseggerweg 48, 8036 Graz, Austria; beate.rinner@medunigraz.at; 6 Department of Biomedical Sciences, University of Padova, Via Ugo Bassi 58/B, 35131 Padova, Italy; samuele.negro@unipd.it

**Keywords:** oncolytic adenovirus, hepatocellular carcinoma, cationic glycopolymers, tumor targeting, cancer therapies, ASGPR

## Abstract

Despite significant advances in chemotherapy, the overall prognosis of hepatocellular carcinoma (HCC) remains extremely poor. HCC targeting strategies were combined with the tumor cell cytotoxicity of oncolytic viruses (OVs) to develop a more efficient and selective therapeutic system. OVs were coated with a polygalactosyl-*b*-agmatyl diblock copolymer (Gal_32_-*b*-Agm_29_), with high affinity for the asialoglycoprotein receptor (ASGPR) expressed on the liver cell surface, exploiting the electrostatic interaction of the positively charged agmatine block with the negatively charged adenoviral capsid surface. The polymer coating altered the viral particle diameter (from 192 to 287 nm) and zeta-potential (from –24.7 to 23.3 mV) while hiding the peculiar icosahedral symmetrical OV structure, as observed by TEM. Coated OVs showed high potential therapeutic value on the human hepatoma cell line HepG2 (cytotoxicity of 72.4% ± 4.96), expressing a high level of ASGPRs, while a lower effect was attained with ASPGR-negative A549 cell line (cytotoxicity of 54.4% ± 1.59). Conversely, naked OVs showed very similar effects in both tested cell lines. Gal_32_-*b*-Agm_29_ OV coating enhanced the infectivity and immunogenic cell death program in HepG2 cells as compared to the naked OV. This strategy provides a rationale for future studies utilizing oncolytic viruses complexed with polymers toward effective treatment of hepatocellular carcinoma.

## 1. Introduction

Hepatocellular carcinoma (HCC) is a primary liver malignancy and the fourth leading cause of death annually [1,2,3], being very aggressive and the most frequent solid tumor of the liver. Globally, 80% of the HCC cases derive from the two most important risk factors, hepatitis B virus (HBV) and hepatitis C virus (HCV) infections [4], while only a minor proportion of HCC can be ascribed to non-viral related factors, such as excessive alcohol consumption, iron overload syndrome, aflatoxin exposure, and also diabetes with subsequent development of non-alcoholic steatohepatitis [5]. Importantly, HBV and HCV infection are responsible for the onset of chronic liver injury, which could further develop into cirrhosis and progress to HCC. In particular, HBV viral proteins hold oncogenic potential, and their DNA can integrate into the host cells genome, directly transforming healthy hepatocytes into cancer cells [6].

Despite several advances in its management, HCC is a highly refractory cancer, resistant to conventional treatments that often provide only marginal survival benefits along with undesirable side effects [7]. Several strategies have therefore been investigated to improve the prognosis of patients affected by HCC [8].

So far, nanotechnology-based approaches to engineering highly specific targeted drug delivery systems able to diagnose, image, and treat HCC have been developed. However, although successful results being obtained with various targeted nanoparticles, their clinical application encounters several hurdles related to their physicochemical instability, poor biopharmaceutical behavior, off-targeting toxicity, uncontrolled drug release profile, etc. [9]. Therefore, there is a high demand to continue the investigation into new therapeutic systems and materials that can offer new opportunities for HCC treatment.

In this realm, a novel and effective approach to treating HCC can be represented by oncolytic virotherapy, which embodies a selective and powerful tool for cancer treatment. Oncolytic adenoviruses (OVs) are able to selectively replicate in tumor cells while sparing the healthy ones [10,11,12,13,14], inducing cytotoxic effects only in cancer cells by selective tumor cell lysis and boosting antitumor immunity [15,16,17,18,19,20]. In addition, OVs have shown encouraging clinical outcomes in terms of safety and efficacy [21], thus entering into the golden age of their utilization since IMLYGIC™ (T-VEC/Talimogene Laherparepvec) was approved, both in the United States and Europe, for the treatment of advanced-stage melanoma. T-VEC is a genetically modified herpes simplex-derived chimeric [22], and its tumor cell specific cytotoxicity is enforced by the virus-driven expression of human granulocyte–macrophage colony-stimulating factor (GM-CSF), which further stimulates the immune system.

The use of OVs for the treatment of HCC has recently gained increased attention due to preliminary studies showing its potential alone or in combination with chemotherapy [23]. For instance, Zou et al. recently reported that OVs associated with quercetin have a synergistic effect on the reduction of HCC growth and tumor volume in mice injected with HuH-7 hepatocyte-derived carcinoma cells [24]. Yoon et al. described the engineering of an OV to preferentially drive OV replication and cell killing in hepatocellular carcinoma (HCC) [25]. OVs have also been used for gene-targeted oncolytic viral therapy in HCC, a novel strategy based on the generation of viral vectors engineered with anticancer genes that selectively infect and kill cancer cells while having no toxic effect on the healthy ones [23].

However, there are some major limitations to the systemic delivery of OVs, including non-specific organ distribution and the production of neutralizing antibodies (NAbs) [26,27]. Therefore, proper OV strategies must be set up to increase OV disease organ disposition, enhance cell targeting and uptake, and reduce systemic immune response.

Among the strategies developed by nanotechnology to enhance the therapeutic performance of nanoparticles for drug delivery, surface decoration with bioactive polymers seems to be an interesting option to bestow cell selectivity and protection from inactivating agents on OVs. Accordingly, polysaccharides could be used to target the asialoglycoprotein receptors (ASGPRs), which are exclusively overexpressed by liver cells and bind galactose with high affinity and deliver OVs to hepatocellular carcinoma cells while reducing the off-organ disposition [28,29]. Therefore, the combination of liver cell targeting conveyed by galactosyl decoration of OVs with the ability of OVs to replicate only in cancer cells results in therapeutic systems with synergistic double selectivity.

Based on this hypothesis, we have designed a novel diblock copolymer constituted by a poly-galactose-based block for ASGPR targeting and a poly-agmatine cationic block for anchoring the copolymer to the negative OV surface. The copolymer was used for coating the adenovirus Ad5/3-D24-ICOSL (OV), and physicochemical features of the polymer-coated OV (PC-OV) were evaluated by dynamic light scattering (DLS), zeta potential analysis, and transmission electron microscopy (TEM). In vitro biological studies were carried out using human hepatoma cell line HepG2, which expresses high levels of ASGPRs, and adenocarcinoma human alveolar basal epithelial A549 cells lacking ASGPR expression. The cell selectivity, cytotoxicity, infectivity, immune-reactivity of PC-OV were comparatively evaluated with respect to naked OV (uncoated), copolymer alone, and a mixture of naked OV and copolymer separately added to the cell culture.

## 2. Materials and Methods

### 2.1. Materials

Acryloyl chloride, Agmatine sulfate, Carbon disulfide, Ethanethiol, 4,4′-azobis(cyanopentanoic acid), N-hydroxyethyl acrylamide, n-Butyl acrylate, 2,2′-azobis[2-(2-imidazolin-2-yl)propane]dihydrochloride (VA-044), 1,4-Dioxane, Dimethylsulfoxide deuterated, Boron trifluoride diethyl etherate, Extravidin-peroxidase, Deuterium oxide, analytical grade solvents, salts, cyanuric chloride, and silicagel (60 Å, particle size 35–70 μm), were obtained from Sigma-Aldrich (St. Louis, MO, USA) and Fisher Scientific (Hampton, NH, USA). α-D-Galactose pentaacetate was purchased from Apollo Scientific (Stockport, UK). Analytical thin-layer chromatography (TLC) was carried out on aluminum sheets coated with silica gel obtained from MACHEREY-NAGEL GmbH & Co. KG (Düren, Germany). Dialysis membranes (MWCO 3.5 and 5 kDa) were provided by Prodotti Gianni S.r.l. (Milano, Italy). Cell culture media and reagents, phosphate-buffered saline (PBS), paraformaldehyde (PFA), and DAPI were purchased from Sigma-Aldrich (St. Louis, MO, USA), Fisher Scientific (Hampton, New Hampshire, USA), or Gibco ThermoFisher Scientific (Waltham, MA, USA). Adenovirus primary antibody 8C4 was obtained from Novus Biologicals. Biotin-SP–conjugated antibody was purchase from Jackson Immunoresearch.

### 2.2. Nuclear Magnetic Resonance

A Bruker DPX400 Ultrashield 400 MHz (Billerica, MA, USA) was used for the monomers and polymers’ characterization. The data were processed with MestReNova software (v6.0.2-5475, 2009, Mestrelab Research S.L., Santiago de Compostela, Spain).

### 2.3. Gel Permeation Chromatography (GPC)

The polymers’ molecular weight and polydispersity index (Đ) were assessed by GPC analysis with Malvern Viscotek TDA302 system (Malvern, UK) equipped with a Refractometer (RI), a Low-Angle Light Scattering (LALS), a Right-Angle Light Scattering (RALS), and a Differential Viscosimeter (Visc) and thermostated at 40 °C equipped with TOSOH G4000 (10 μm, 7.8 × 300 mm) and G3000 (7 μm, 7.8 × 300 mm) PWXL columns in series eluted with 0.4 M ammonium acetate buffer pH 4.5. Data acquisition was performed by Omnisec 5.1 software using the pullulan standard for calibration.

### 2.4. Cell Culture

HepG2 (hepatocellular cancer cell line) and A549 (lung cancer cell line) were purchased from the American Type Culture Collection (ATCC, USA). Cells were cultured at 37 °C, 5% CO_2_, and humidified atmosphere using Dulbecco’s modified Eagle medium (DMEM, Lonza, Switzerland) supplemented with 10% fetal bovine serum (FBS, Gibco Laboratories, USA), 100 U/mL penicillin, 100 µg/mL streptomycin (Gibco Laboratories), and 2 mM L-glutamine (Gibco Laboratories) (complete medium). Juvenile fibroblasts were kindly provided by Dr. Rinner from the Medical University of Graz and were cultured at 37 °C, and 5% CO_2_ in a humidified atmosphere using DMEM (Lonza, Switzerland) supplemented with 2 mM L-glutamine (Gibco Laboratories) and 10% fetal bovine serum (FBS, Gibco Laboratories, USA).

### 2.5. Synthesis of 4-(4,6-Dimethoxy-1,3,5-triazin-2-yl)-4-methylmorpholinium Chloride (DMTMM) (***2***)

DMTMM was synthesized according to a two-step procedure as described by Cronin et al. [30] and Kunishima et al. [31] (Scheme S1, Figures S1–S4).

#### 2.5.1. Synthesis of 2-chloro-4,6-dimethoxy-1,3,5-triazine (CDMT) (**1**)

Briefly, methanol (MeOH) (6.75 mL), MilliQ water (659 µL), and Na_2_CO_3_ (3.42 g, 40,7 mmol) were mixed and cooled to 10–15 °C. Cyanuric chloride (2.5 g, 13.6 mmol) was added under stirring, and the resulting mixture was warmed to 35 °C. The reaction mixture was stirred for 12 h until the complete disappearance of the starting material was confirmed by ^1^H NMR analysis. Then, 5 volumes of DI water were added, and the resulting mixture was further stirred for 30 min at room temperature. The product was isolated by filtration, and the solid residue was washed with DI water. The final product was dried under vacuum at 30 °C to yield the intermediate CDMT (1.279 g, 7.4 mmol, 54.7% yield) that was used for the next step without further purification. Indeed, the reaction was carried out according to Cronin et al. [30], conditions that minimize the formation of side product 2,4,6-trimethoxy-1,3,5-triazine derivative (1–2%), which would not react in the next step can be removed according to the purification procedure described below.

^1^H NMR (400 MHz, CDCl_3_, δ): 4.07 (s, 6H, OC*H*_3_).

^13^C{^1^H}NMR (100 MHz, CDCl_3_, δ): 56.20 (2C, O*C*H_3_), 172.24 (2C, *C*O), 172.91 (1C, *C*Cl).

#### 2.5.2. Synthesis of 4-(4,6-Dimethoxy-1,3,5-triazin-2-yl)-4-methylmorpholinium Chloride (DMTMM) (**2**)

CDMT (750 mg, 4.28 mmol) and N-methylmorpholine (NMM) (433 mg, 4.28 mmol, 471 µL) were dissolved in tetrahydrofuran (THF) (17 mL) and left under stirring at room temperature for 30 min. The formation of a white precipitate was observed that was collected by filtration, rinsed with THF, and dried under reduced pressure to yield a white powder (850 mg, 3.07 mmol, 71.93%).

^1^H NMR (400 MHz, MeOH-*d_4_*, δ): 3.54 (*s*, 3H, NC*H*_3_), 3.76–3.95 (*m*, 4H, NC*H_2_*CH_2_O), 4.02–4.12 (*m*, 2H, NC*H_2_*CH_2_O), 4.18 (*s*, 6H, OC*H_3_*), 4.49–4.60 (*m*, 2H, NCH_2_C*H_2_*O), 4.79–4.85 (*m*, 2H, NCH_2_C*H_2_*O).

^13^C{^1^H}NMR (100 MHz, MeOH-*d_4_*, δ): 56.45 (1 C, N*C*H_3_), 57.55 (2C, O*C*H_3_), 61.41 (2C, NCH_2_*C*H_2_O), 63.20 (2C, N*C*H_2_CH_2_O), 171.93 (1C, N*C*N^+^), 175.48 (2C, N*C*OCH_3_).

### 2.6. Synthesis of 4-cyano-4-(ethylsulfanylthiocarbonylsulfanyl)pentanoic Acid RAFT Agent (***4***)

The synthesis of the RAFT agent 4-cyano-4-(ethylsulfanylthiocarbonylsulfanyl)pentanoic acid was performed in a two-step reaction according to the procedure described by Truong et al. [32] (Scheme S2, Figures S5 and S6).

#### 2.6.1. Synthesis of bis(ethylsulfanylthiocarbonyl)disulfide (**3**)

In a 2-neck round bottom flask, 9.6 mL of a 0.5 mg/mL NaOH aqueous solution (0.12 mol) was added dropwise over 5 min under nitrogen atmosphere to a stirred solution of ethanethiol (7.4 mL, 0.10 mol) in a mixture of water/acetone 20:5 (25 mL) cooled on ice. Carbon disulfide (CS_2_) (8.37 g, 0.11 mol) was added to the reaction with cooling and constant stirring over a 1-h period, and the reaction was stirred until total consumption of CS_2_, which was checked by extended range ^13^C NMR. Potassium ferrocyanide (36.21 g, 0.11 mol) was immediately added and stirred for 30 min at 0 °C until the solution turned dark yellow, and a precipitate was observed. The reaction mixture was extracted with DCM, and the organic layer was then washed twice with aqueous saturated NaHCO_3_ (2 × 30 mL), twice with distilled water (2 × 30 mL), and dried with MgSO_4_. After filtration, the solvent was removed under reduced pressure to yield bis(ethylsulfanylthiocarbonyl)disulfide as a crude orange oil residue (9.5 g, 0.035 mol, 70% yield). The crude product was used for the next step reaction without further purification.

^1^H NMR (400 MHz, CDCl_3_, δ): 1.36 (*t*, 3H, *J* = 7.5 Hz, C*H_3_*,), 3.31(*q*, 2H, *J* = 7.5 Hz, C*H_2_*).

^13^C{^1^H}NMR (100 MHz, CDCl_3_, δ): 12.5 (1C, *C*H_3_), 32.7 (1C, *C*H_2_), 221.4 (1C, -S*C*(S)S-).

#### 2.6.2. Synthesis of 4-cyano-4-(ethylsulfanylthiocarbonylsulfanyl)pentanoic Acid (**4**)

Five grams of crude bis(ethylsulfanylthiocarbonyl)disulfide (0.021 mol) was dissolved in ethanol, and 4,4′-azobis(cyanopentanoic acid) (8.30 g, 0.029 mol) was added. The reaction mixture was degassed by nitrogen bubbling for 10 min, heated at 60 °C, and left under stirring at this temperature overnight. The reaction was then cooled to room temperature, and the solvent was evaporated under reduced pressure. The final product was purified by column chromatography on SiO_2_ and was eluted using a gradient of DCM/EtOAc from 10:0 to 9:1. Two column purifications were necessary to remove traces of starting material. The solvent was removed under reduced pressure to yield the RAFT agent 4-cyano-4-(ethylsulfanylthiocarbonylsulfanyl)pentanoic acid (1.8 g, 8 mmol, 38% yield) as a yellow powder.

^1^H NMR (400 MHz, CDCl_3_, δ): 1.36 (*t*, 3H, *J* = 7.4, Hz C*H_3_*CH_2_,), 1.89 (*s*, 3H, C*H_3_*C), 2.33–2.60 (*m*, 2H, C*H_2_*CH_2_COOH), 2.64–2.73 (*m*, 2H, CH_2_C*H_2_*COOH), 3.33 (*q*, 2H, *J* = 7.4 Hz, C*H_2_*).

^13^C{^1^H}NMR (100 MHz, CDCl_3_, δ): 12.85 (1C, *C*H_3_CH_2_), 25.02 (1C, *C*H_3_C), 29.48 (1C, CH_2_*C*H_2_COOH), 31.55 (1C, *C*H_2_CH_2_COOH), 33.69 (1C, CH_3_*C*H_2_), 46.37 (1C, CH_3_*C*CN), δ 119.03 (1C, *C*N),176.18 (1C, CH_2_*C*OOH), 216.77 (1C, S*C*(S)S).

ESI-TOF *m/z*: [M-Na]^+^ calcd for [C_9_H_13_NO_2_S_3_Na]^+^ 286.0001, found 285.9994 (100.0%).

### 2.7. Synthesis of O-β-D-galactopyranosyloxyethyl Acrylamide (***6***)

The synthesis of α-D-galactopyranosyloxyethyl acrylamide was performed according to the procedure described by Martinez-Pomares et al. [33] (Scheme S3, Figures S7 and S8).

#### 2.7.1. Synthesis of 2,3,4,6-O-tetraacetyl-D-galactopyranosyloxyethyl Acrylamide (**5**)

β-D-Galactose pentaacetate (25 g, 64.10 mmol) was dissolved in CH_2_Cl_2_ (50 mL), and *N*-hydroxyethyl acrylamide (11.05 g, 96 mmol, 10 mL) was added under stirring. BF_3_Et_2_O (13.65 g, 96 mmol, 12 mL) was added dropwise over 1 h to the reaction mixture pre-cooled on an ice bath. The reaction was left under stirring at room temperature for 48 h. At scheduled times (every 24 h), the progress of the reaction was monitored by ^13^C NMR analysis in DMSO-*d_6_* of samples withdrawn from the reaction solution by checking the shift of the anomeric carbon from 89 to 100. At full conversion, the reaction mixture was poured into saturated aqueous NaHCO_3_. Finally, the organic phase was washed with DI water (2 × 125 mL), dried over MgSO_4_, and the solvent evaporated under reduced pressure. The crude product was purified by flash chromatography (Φ = 5 cm, h = 15 cm) (Silica gel 60 Å, particle size 35–70 µm, gradient elution from 100% Et_2_O to 100% EtOAc). Fractions were analyzed by TLC (100% Et_2_O as eluent), and the appropriate were concentrated to yield compound 2,3,4,6-O-tetraacetyl-D-galactopyranosyloxyethyl acrylamide as white crystals (6.623 g, 14.86 mmol, 23.2% yield).

^1^H NMR (400 MHz, DMSO-*d_6_*, *δ*): 1.91 (*s*, 3H, C*H*_3_), 1.99 (*s*, 3H, C*H*_3_), 2.01 (*s*, 3H, C*H*_3_), 2.12 (*s*, 3H, C*H*_3_), 3.29 (*dt*, *J* = 7.0, 3.5 Hz, 2H, C*H*_2_NH), 3.54–3.66 (*m*, 1H, CHC*H*H), 3.69–3.80 (*m*, 1H, CHCH*H*), 3.97–4.08 (*m*, 2H, C*H*_2_CH_2_NH), 4.20 (*dd*, *J* = 7.0, 6.2 Hz, 1H, C*H*), 4.73 (*d*, *J* = 1.3 Hz,1H, C*H* anomeric), 4.94 (*dd*, *J* = 9.9 Hz, 1H, C*H*), 5.15 (*dd*, *J* = 3.5, 1.6 Hz, 1H, C*H*) 5.26 (*d*, *J* = 9.9, 3.5 Hz, 1H, C*H*), 5.58 (*dd*, *J* = 10.1, 2.3 Hz, 1H, CH=C*H*H), 6.08 (*dd*, *J* = 17.1, 2.3 Hz, 1H, CH=CH*H*), 6.23 (*dd*, *J* = 17.1, 10.1 Hz, 1H, C*H*=CH_2_), 8.12 (*t*, *J* = 5.4 Hz, 1H, N*H*).

^13^C{^1^H}NMR (100 MHz, DMSO-*d_6_*, *δ*): 20.79 (1C, *C*H_3_), 20.86 (1C, *C*H_3_), 20.95 (2C, *C*H_3_), 39.08 (1C, *C*H_2_NH), 61.67 (1C, CH*C*H_2_), 67.83 (1C, *C*H_2_CH_2_NH), 68.25 (1C, *C*HOC=O), 69.02 (1C, *C*HOC=O), 70.46 (1C, *C*HOC=O), 70.71 (1C, *C*HCH_2_OC=O), 100.34 (1C, *C* anomeric), 125.50 (1C, CH=*C*H_2_), 132.32 (1C, *C*H=CH_2_), 165.12 (1C, NH*C*=O), 169.61 (1C, *C*=OCH_3_), 169.97 (1C, *C*=OCH_3_), 170.37 (1C, *C*=OCH_3_), 170.40 (1C, *C*=OCH_3_).

ESI-TOF *m/z:* [M-H]^+^ calcd for [C_19_H_28_NO_11_]^+^ 446.428; found 446.1736.

FT-IR *ν:* 3262, 3063, 2882, 1752, 1616, 1365, 1217, 1172, 1134, 1093, 1045, 934, 901, 812, 737, 601 cm^−1^.

#### 2.7.2. Deprotection of 2,3,4,6-O-D-galactopyranosyloxyethyl Acrylamide to Give D-galactopyrano- Syloxyethyl Acrylamide (**6**)

2,3,4,6-O-tetraacetyl-D-galactopyranosyloxyethyl acrylamide (6.623 g, 14.86 mmol) was dissolved in 73 mL of a KOH (83 mg, 1.486 mmol) methanolic solution. The mixture was left under stirring at room temperature for 48 h and passed through a short pad of silica (silicagel 60 Å, particle size 35–70 µm, eluent 100% CH_3_OH). The organic phase was concentrated under pressure to isolate the D-galactopyranosyloxyethyl acrylamide (**2**) (3.9 g, 14.06 mmol, 95.28% yield).

^1^H NMR (400 MHz, DMSO-*d_6_,δ*): 3.26–3.34 (*m*, 5H, C*H*OH, C*H_2_*CH_2_NH, C*H_2_*NH), 3.44–3.57 (*m*, 3H, C*H*OH, C*H*_2_OH), 3.62 (m, 1H, C*H*OH), 3.75 (*dt*, 1H, *J* = 10.4, 5.8 Hz, C*H*CH_2_OH), 4.09 (*d*, 1H, *J* = 7.1 Hz, C*H* anomeric), 4.39 (*bs*, 1H, *J* = 3.0 Hz, O*H*,), 4.64 (*bs*, 1H, O*H*) 4.51 (*bs*, 1H, O*H*), 4.86 (*bs*, 1H, O*H*), 5.58 (*dd*, *J* = 10.1, 2.2 Hz, 1H, CH=C*H*H), 6.08 (*dd*, *J* = 17.1, 2.2 Hz, 1H, CH=CH*H*), 6.24 (*dd*, *J* = 17.1, 10.1 Hz, 1H, C*H*=CH_2_), 810 (*t*, *J* = 5.4 Hz, 1H, N*H*).

^13^C{^1^H}NMR (100 MHz, DMSO-*d_6_*, *δ*): *δ* 48.62 (1C, *C*H_2_NH), 60.51 (1C, *C*H_2_OH), 67.73 (1C, *C*H_2_CH_2_NH), 6816 (1C, *C*HOH), 70.58 (1C, *C*HOH), 73.31 (1C, *C*HOH), 75.30 (1C, *C*HCH_2_OH), 103.78 (1C, *C* anomeric), 125.21 (1C, CH=*C*H_2_), 131.70 (1C, *C*H=CH_2_), 164.70 (1C, NH*C*O).

ESI-TOF *m/z:* [M-Na]^+^ calcd for [C_11_H_19_NO_7_Na]^+^ 300.2597; found 300.1092.

FT-IR *ν* 3324, 2933, 2344, 1647, 1559, 1250, 1076, 893, 779 cm^−1^.

### 2.8. Synthesis of Agmatine Acrylamide (Agm) (***7***)

Agmatine Acrylamide (Agm) was synthesized according to the procedure described by Algotsson M. et al. [34] with slight modifications (Scheme S4, Figures S9 and S10).

Agmatine Sulfate (2 g, 8.76 mmol) was dissolved in a 30 mM solution of K_2_CO_3_ (16 mL, 0.48 mmol) under stirring, and the reaction mixture was cooled at 0 °C in an ice bath. Acryloyl chloride (7.12 mL, 87.6 mmol) was added in 350 µL aliquots. The pH of the solution was monitored and maintained in the range of 8 to 9 by the addition of the required amount of 2 M NaOH solution. The reaction was monitored by TLC using 100% methanol (MeOH) as eluent until the disappearance of the violet spot visualized by ninhydrin staining (0.2% *w/v* ethanolic solution). Subsequently, the mixture was acidified to pH 2 with 1 M HCl. The side-product acrylic acid was removed by extraction with 10 × 15 mL of chloroform (CHCl_3_), and the purification was monitored by TLC using 100% methanol (MeOH) until the complete disappearance of the acrylic acid spot in the organic phase. The aqueous phase was collected, neutralized with 1 M NaOH, and traces of solvent were removed under vacuum. The aqueous phase containing the product was freeze-dried, re-dissolved in MeOH, and passed through a short pad of silica to remove salts (Silica gel 60 Å, particles size 35–70 μm). The solvent was removed under reduced pressure, and the pure product was re-dissolved in DI water and recovered by lyophilization. Acryloyl agmatine was obtained as a white powder (1.2 g, 6.5 mmol, 74.2% yield).

^1^H NMR (400 MHz, D_2_O, δ): 1.59 (*m*, 4H, NHCH_2_C*H_2_*C*H_2_*CH_2_), 3.19–3.28 (*m*, 4H, NHC*H_2_*CH_2_CH_2_C*H_2_*NH), 5.74 (*dd*, *J* = 9.8, 1.8 Hz, 1H CH*H*=CH), δ 6.16 (*dd*, *J* = 17.1, 1.8 Hz, 1H, C*H*H=CH); 6.25 (*dd*, *J* = 17.1, 9.8 Hz, 1H, CH_2_=C*H*).

^13^C{^1^H}-NMR (100 MHz, D_2_O, δ): 25.30 (1C, NHCH_2_*C*H_2_CH_2_CH_2_NH), 25.56 (1C, NHCH_2_CH_2_*C*H_2_CH_2_NH), 38.83 (1C, NH*C*H_2_), 40.86 (1C, *C*H_2_NH), 127.21 (1C, *C*H_2_=CH), 130.04 (1C, CH_2_=*C*H), 156.74 (1C, NH_2_*C*NH), 168.56 (1C, NH*C*=O).

ESI-TOF *m/z*: [M-H]^+^ calcd for [C_8_H_17_N_4_O]^+^ 185.13; found 185.140.

FT-IR ***ν*** = 3314, 2875, 1648, 1605, 1264, 1087, 610, 553 cm^−1^.

### 2.9. Synthesis of Gal_32_-b-Agm_29_ Cationic Diblock Copolymer (***9***)

Gal_32_-*b*-Agm_29_ copolymer was synthesized by Fast RAFT polymerization according to the procedure reported by Gody et al. [35] and modified by Mastrotto et al. [33,36].

Briefly, D-Galactopyranosyloxyethyl acrylamide (Gal, 157 mg, 0.57 mmol) was dissolved in MilliQ water (157 μL), placed in a tube equipped with a magnetic follower, and RAFT agent (4.7 mg, 17.7 µmol; 31.7 µL of 150 mg/mL stock solution in dioxane) was added. The tube was then placed in an ice bath, and freshly prepared VA-044 (0.11 mg, 0.36 µmol; 11.46 µL of 10 mg/mL stock solution in MilliQ water) was added to the reaction mixture under stirring. The tube was sealed with a rubber septum and deoxygenated by gentle Argon bubbling for 10 min. The polymerization was started by placing the tube in an oil bath preheated at 60 °C. The reaction was monitored by ^1^H NMR in DMSO-*d*_6_, analyzing samples withdrawn from the polymerization mixture at 2 h. Sequential addition of VA-044 solution was made until the conversion percentage was >90%. The generated macro-CTA Gal_32_ (**8**) was finally characterized by ^1^H NMR.

^1^H NMR (400 MHz, DMSO-*d_6_*, δ, ppm): 7.51 ppm (*br*, 1H, NH), 4.44 ppm (*d,* 1H, O-CH *anomeric*), 4.16–5.05 ppm (*m*, 4H, C-OH *sugar*), 0.75–2.13 ppm (*m*, 3H, CHCH_2_ *polymer backbone*).

Conversion: 100%; *M*_n,th_ = 9.1 kDa; *DP* = 32.

Agmatine acrylamide (104.5 mg, 0.567 mmol) was dissolved in MilliQ water (227 μL) in a separate tube and then transferred to the reaction vessel (theoretical molar ratio Gal/Agm 1:1). The reaction vessel was placed in an ice bath and a freshly prepared VA-044 initiator (0.11 mg, 0.36 µmol; 11.46 µL of a 10 mg/mL stock solution in MilliQ water) was added to the reaction mixture under stirring. The reaction mixture was degassed by Argon bubbling for 10 min. The polymerization was started by placing the tube in an oil bath preheated at 60 °C. After 2 h, the conversion was monitored by ^1^H NMR in DMSO-*d_6_* on a sample withdrawn from the reaction vessel, and the polymerization was considered complete when Agm conversion was >80%; otherwise, a second addition of the VA-044 initiator was made. The polymer was then dialyzed against MilliQ water for 48 h using a 3.5 kDa MWCO dialysis membrane to remove the unreacted monomers. The product was freeze-dried, and the copolymer Gal_32_-*b*-Agm_29_ (**9**) (163 mg, 11.2 µmol, 65.1% yield) was recovered as a yellowish powder and analyzed by ^1^H NMR and GPC.

^1^H NMR (400 MHz, DMSO-*d*_6_, δ, ppm): 6.78–8.42 ppm (*bm*, 5H, NH galactose, NH agmatine, NH guanidyl group), 4.44 ppm (*d*, 1H, O-CH anomeric), 4.16–5.05 ppm (*m*, 4H, C-OH sugar), 0.66–2.33 ppm (*bm*, 10H, CHCH_2_ polymer backbone, N-CH_2_-CH_2_-CH_2_-CH_2_ agmatine).

Conversion: 90%; *M*_n_,_th_ = 14.5 kDa; *DP* = 61; *M_n_*_(GPC, aqueous)_ = 21.9 kDa; Đ _(GPC, aqueous)_ = 1.4.

Gal_255_ (**10**) homopolymer used for the competition assay was prepared as described above for Gal_32_ using a [Gal]:[RAFT Agent]:[VA-044] molar ratio of 260:1:0.02 and purified at the end of the polymerization reaction by dialysis against MilliQ water for 48 h using a 3.5 kDa MWCO dialysis membrane to remove the unreacted monomer. The product was recovered by freeze-drying (358.4 mg, 5.05 mmol, 80% yield).

Conversion: 98%; *M*_n_,_th_ = 70.9 kDa; *DP* = 250; *M_n_*_(GPC, aqueous)_ = 82.2 kDa; Đ _(GPC, aqueous)_ = 1.09.

### 2.10. Synthesis of Cy5 labeled Gal_32_-b-Agm_29_ (Cy5-Gal_32_-b-Agm_29_) (***11***)

Gal_32_-*b*-Agm_29_ (30 mg, 2.07 µmol) was dissolved in 500 µL of 50 mM MES (2-(*N*-morpholino)ethanesulfonic acid) buffer pH 6.5. One hundred and forty-eight microliters of a 10 mg/mL Cyanine-5-amine (Cy5-NH_2_) solution in DMSO was then added, followed by 27.5 µL of a freshly prepared 20 mg/mL DMTMM stock solution in MES. The reaction was left under stirring for 16 h at room temperature. The solution was then transferred into a 5 kDa MWCO dialysis bag and dialyzed against 5 L of DI water for 3 days with at least two water exchanges per day. Finally, Cy5-Gal_32_-*b*-Agm_29_ (25.4 mg, 16.8 µmol, 81.2%) was isolated as a light blue powder by lyophilization. The reaction scheme is available in the Supplementary Materials (Scheme S5).

### 2.11. Ad5/3-D24-ICOSL Oncolytic Adenovirus Production and Characterization

The adenovirus vector was kindly provided by Dr. Kuryk from the NIPH-NIH (Poland) for the purpose of this experiment. The vector was generated and amplified using standard adenovirus preparation techniques [37,38]. Briefly, the Ad5 WT virus was used as a backbone for the construct (Genbank #AY339865), and the following genetic modifications were made: (i) deletion of 24 bp in the E1A conserved region, (ii) insertion of the inducible costimulatory ligand (ICOSL) [39,40] was placed under the exogenous promoter in the E3 region. Additionally, the fiber knob was modified (AdV5/3 chimeric). Shuttle plasmids were constructed and contained modifications in E1A, E3, and fiber regions. Subsequently, the shuttle plasmids were digested with SfiI and ligated to each other. The ligation products were packed into phage lambda and infected E. coli. The selection of ampicillin and kanamycin-resistant clones was performed and was followed by DNA transfection by linearization of the cosmid DNA with PacI or SwaI. Linear cosmid was transfected into A549 helper cells. Finally, plaques were harvested, and virus identity was confirmed by restriction enzyme assays. Amplification of the virus was done in A549 cells, followed by purification on two CsCl gradients, dialysis against an isotonic buffer (default = GTS buffer: 2.5% glycerol, 25 mM NaCl, 20 mM Tris-HCl, pH 8.0). The virus suspension was filtered through a low-protein binding 0.22 μm filter. The viral particle concentration was determined by OD_260_-reading, and a standard TCID_50_ (tissue culture infectious dose 50) assay was performed to determine infectious particle titer [41,42].

### 2.12. Formulation of Unlabeled and Fluorescently Labeled Gal_32_-b-Agm_29_ Coated Ad5/3-D24-ICOSL Adenovirus

Gal_32_-*b*-Agm_29_ polymer-coated Ad5/3-D24-ICOSL oncolytic adenovirus (hereinafter referred as “PC-OV”) was prepared by mixing virus particle (VP) stock solution (1 µL, 1 × 10^12^ VP/mL) in PBS, with Gal_32_-*b*-Agm_29_ (1 µL, 10 mg/mL solution) block copolymer stock solution in MilliQ water. The mixture was left at room temperature for 15 min and then diluted to 800 µL in the appropriate buffer/medium according to the experiment performed, as described in the following paragraphs.

Fluorescently labeled VPs were prepared using a 70:30 mixture of Gal_32_-*b*-Agm_29_ and Cyanine 5-labeled Gal_32_-*b*-Agm_29_ (Cy5- Gal_32_-*b*-Agm_29_).

### 2.13. Size and Zeta Potential Analyses

The mean particle diameter, polydispersity (PDI), and zeta potential (ZP) of naked OV (uncoated copolymer Ad5-D24-ICOSL) or PC-OV were determined by Dynamic Light Scattering (DLS) using Malvern Instrument Ltd. Zetasizer Nano ZS (Malvern, UK) at a constant scattering angle of 173° and 25 °C. Formulations were prepared by diluting naked OV or PC-OV suspensions prepared as described in ”Formulation of unlabeled and fluorescently labeled Gal_32_-b-Agm_29_ coated Ad5/3-D24-ICOSL adenovirus”.

### 2.14. Electron-Microscope Characterizations

The morphology and structure of naked OV and PC-OC were examined by transmission electron microscopy (TEM) using the Tecnai G2 microscope (FEI). Samples, prepared as described in “Formulation of unlabeled and fluorescently labeled Gal_32_-b-Agm_29_ coated Ad5/3-D24-ICOSL adenovirus”, were deposited on a small holey carbon-coated support grid (400 mesh), and the solvent was allowed to dry at room temperature. Samples were negatively stained with 1% *w/v* aqueous uranyl acetate solution prior to imaging. TEM images were captured by rotating the sample from +60° to −60° and collected every 2° of rotation.

### 2.15. Evaluation of Expression Level of the Coxsackievirus and Adenovirus Receptor (CAR) and Desmoglein-2 (DSG2) in Hepatocellular Carcinoma and Lung Cancer Cells

CAR and DSG2 expression levels in HepG2 and A549 cells were assessed by staining with primary mouse monoclonal anti-CAR antibody (Santa Cruz Biotech, Dallas, TX, USA) and mouse monoclonal anti-DSG2 antibody (Abcam, Cambridge, UK), respectively, and subsequently with 1:2000 diluted Alexa-Fluor^®^ 488- conjugated secondary antibody (Abcam, Cambridge, UK) for flow cytometry analysis (BD FACSCantoTM II, Franklin Lakes, NJ, USA). The experiment was performed in duplicate.

### 2.16. Cell Cytotoxicity Studies

HepG2 and A549 cancer and Juvenile fibroblast cell lines were seeded at a density of 1 × 10^4^ cells/well in 96-well plates and maintained under standard growth conditions. After 24 h, cells were treated with 100 μL of cell culture medium containing (i) naked OV; (ii) Gal_32_-*b*-Agm_29_ alone; (iii) PC-OV prepared as described in “Formulation of unlabeled and fluorescently labeled Gal_32_-b-Agm_29_ coated Ad5/3-D24-ICOSL adenovirus”; (iv) OV and Gal_32_-*b*-Agm_29_ were separately added to the cell culture medium immediately before addition to the cells (hereinafter referred as Gal_32_-*b*-Agm_29_ /OV mix). All samples added to the cells contained equivalent concentrations of OV (1.25 × 10^10^ VP/mL) and Gal_32_-*b*-Agm_29_ (12.5 μg/mL). After 72 h, cell viability was assessed by MTS assay according to the manufacturer’s protocol (Cell Titer 96 Aqueous One Solution Cell Proliferation Assay; Promega, Nacka, Sweden) using a 96-wells plate spectrophotometer Varioskan Flash Multimode Reader (Thermo Scientific) set at λ = 490 nm. The absorbance value for untreated cells was set as 100% viability (control), and the viability of treated cells was expressed as a percentage of the absorbance with respect to the absorbance value of the control. Three independent experiments were performed in triplicate for each condition.

### 2.17. Cell Competition Studies

HepG2 cells were seeded in 12-well plates at a seeding density of 2 × 10^5^ cells/well in 2 mL of complete medium and grown at 37 °C, 5% CO_2_, and humidified atmosphere. After 24 h, the culture medium was replaced with 1 mL of 250 µg/mL Gal_255_ polymer solution in cell culture medium or cell culture medium alone in the appropriate well. After 1 h incubation, Cy5- Gal_32_-*b*-Agm_29_ coated Ad5/3-D24-ICOSL adenovirus (2 μL, 0.5 × 10^12^ VP/mL and 5 mg/mL polymer concentration) prepared as described in the paragraph “Formulation of unlabeled and fluorescently labeled Gal_32_-b-Agm_29_ coated Ad5/3-D24-ICOSL adenovirus”, was added to the appropriate wells. Control cells were added to an equal volume of cell culture medium. Cells were further incubated for 1 h, and then the medium was discharged; cells were rinsed 3 × 1 mL PBS and harvested by trypsin treatment (100 μL, 0.03% *w/v* solution in PBS). Cell suspensions were immediately diluted with 200 μL of PBS followed by fixation with 100 μL of a 4% *v/v* PFA solution in PBS and stored in the dark at 4 °C until FACS analysis. Cells were analyzed by a BD FACSAriaIIITM (BD, Franklin Lakes, USA) flow cytometer, and at least 1 × 10^4^ events per sample were recorded. The mean fluorescence intensity and the percentage of positive cells were detected on the Cy5 channel (λ_ex_ 633, λ_em_ 660/20). Untreated cells served as negative controls. Data were analyzed with FlowJo software (v10.2, 2016, Becton, Dickinson and Company, Ashland, OR, USA).

### 2.18. Determination of the Infectious Titer

The determination of the infectivity was based on the detection of adenovirus hexon protein expression by immunostaining the infected cells. HepG2 and A549 cells were seeded at a density of 2 × 10^5^ cells/well in 24-well plates and maintained under standard growth conditions. Cells were treated with 500 μL/well of formulations containing naked OV, PC-OV, and Gal_32_-*b*-Agm_29_ /OV mix samples prepared as described in “Cell cytotoxicity studies”. After 48 h of incubation, supernatants were aspirated, and the cells fixed by incubation for 10 min with 250 µL of ice-cold methanol per well. Cells were then rinsed with 3 × 300 µL of 1% *w/v* solution of bovine serum albumin in PBS and incubated with 250 µL of mouse monoclonal anti-hexon primary antibodies (1:2000 dilution in a 1% BSA in PBS) for 1 h at room temperature in the dark. Afterward, cells were washed 3 × 300 µL with 1% BSA in PBS and incubated with 250 µL of the secondary antibody (Biotin-SP–conjugated antibody), 1:500 dilution with 1% BSA in PBS for 1 h at room temperature in the dark. Finally, cells were rinsed with 1% BSA in PBS (3 × 300 µL) and incubated in the dark with 250 µL of Extravidin-peroxidase (1:200 dilution in 1% BSA in PBS) for 30 min at room temperature before being visualized by treatment with 250 µL of 1 × 3,3’-diaminobenzidine tetrahydrochloride (DAB) solution, according to the manufacturer’s instructions. For each of five replicates (wells), five images of non-overlapping fields were acquired using an AMG EVO XL microscope (ThermoFisher Scientific, Waltham, MA, USA), and positive stained cells, detected as brown spots, were counted. Infectivity data are presented as the average number of positive cells per well. Three independent experiments were performed in triplicate for each condition.

### 2.19. ATP and HMGB1 Release

HepG2 and A549 cell lines were seeded in triplicate onto 96-well plates at 1 × 10^4^ cells/well and infected with naked OV, Gal_32_-*b*-Agm_29_/OV mix, and PC-OV samples prepared as described in “Cell cytotoxicity studies”. Three independent experiments were performed in triplicate for each condition.

#### 2.19.1. ATP Release

Forty-eight hours after cell infection, supernatants were collected and analyzed with the ATP Determination Kit according to the manufacturer’s protocol (Promega, Madison, WI, USA) by luminometric analysis (Varioskan Flash, ThermoFisher Scientific, Waltham, MA, USA). Untreated cells were used as control. The percentage of released ATP was calculated according to the following equation:

(1) $$\mathrm{ATP}\%=100-\frac{{\mathrm{RLU}}_{\mathrm{sample}}}{{\mathrm{RLU}}_{\mathrm{control}}}\times 100$$

where RLU is the relative light unit.

#### 2.19.2. HMGB-1 Release

Seventy-two hours after cell infection, supernatants were collected, and HMGB1 was measured with an Elisa kit according to manufacturer instructions (MBL International, Woburn, MA) and referred to a calibration curve obtained with serial dilution of standard HMGB1.

#### 2.19.3. Analysis of Apoptotic and Necrotic Cells

A549 and HepG2 cells were plated into 6-well plates at a density of 2 × 10^5^ cells/well. Cells were treated with naked OV, Gal_32_-*b*-Agm_29_ alone, PC-OV, and Gal_32_-*b*-Agm_29_/OV mix samples at the concentrations described in “Cell cytotoxicity studies”. The amount of apoptotic and necrotic cells was measured at 24 h post-treatment with a TACS Annexin V-FITC kit (Trevigen Inc., Gaitherburg, MD, USA) and a BD FACSCantoTM II (Franklin Lakes, NJ, USA) flow cytometer according to the manufacturer instructions. Three independent experiments were performed in triplicate for each condition.

### 2.20. Statistical Analysis

Statistical significance was analyzed by using one-way ANOVA with Tukey’s Multiple Comparison test, and nonparametric Mann–Whitney test, and Student’s t-test. All statistical analysis, calculations, and tests were performed using GraphPad Prism (v7.0, 2018, GraphPad Software, San Diego, CA, USA).

## 3. Results and Discussion

Based on previous evidence demonstrating that cationic peptides can bestow cell targeting on oncolytic adenoviruses, we focused on improving the antitumor efficacy of oncoviruses (OVs) for HCC treatment by OV coating with cationic galactosylated block copolymers [43,44].

Galactose-mediated delivery of anticancer drugs to the liver exploits the asialoglycoprotein receptors (ASGPRs), which are expressed in the liver cells and not in other human cells [45,46]. Therefore, the OV decoration with galactosylated polymers may yield polymer/OV complexes with liver cell targeting that synergistically combined with the intrinsic ability of OVs to replicate only in tumor cells while sparing healthy cells, may result in extraordinary therapeutic selectivity for HCC. Accordingly, a galactosylated diblock copolymer, containing a poly-galactose block for HCC targeting and a positively charged poly-agmatine block for OV anchoring was designed for adenoviruses coating. The positively charged poly-agmatine was introduced in the copolymer structure to physically anchor the copolymer through electrostatic interactions to the negatively charged surface of the adenoviral capsid.

The adenovirus Ad5/3-D24-ICOSL was chosen for its great potential for oncolytic virotherapy. Indeed, Ad5/3-D24-ICOSL has modifications that can contribute to its safety [33] and efficacy against HCC. In particular, the deletion of 24 bp in the E1A gene confers cancer cell restricted replication in tumor cells with an altered Rb pathway and incorporates transgene ICOSL into the E3 region of the adenovirus genome, which promotes systemic antitumor immunity and mediates the oncolysis of the tumor mass. OV is taken up by cells through DSG2 and CAR receptors that are expressed by a variety of tumor cells, including HCC cells. The coating with galactosylated copolymer has been designed to restrict the OV selectivity and internalization into ASGPR expressing cells, such as HCC cells.

### 3.1. Synthesis of Gal_32_-b-Agm_29_ Cationic Block Copolymer

**Scheme 1:**
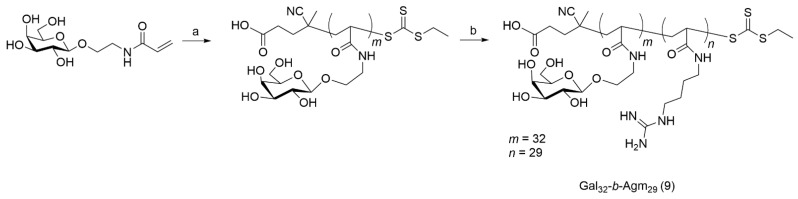
Synthesis of Gal_32_-*b*-Agm_29_ block copolymer (**9**). *Reagents and conditions*: (**a**) RAFT Agent, VA-044, water/dioxane 80:20 *v/v*, Ar atmosphere, 60 °C, 2 h; (**b**) agmatine acrylamide, VA-044, water/dioxane 80:20 *v/v*, Ar atmosphere, 60 °C, 2 h.

The synthesis of Gal_32_-*b*-Agm_29_ block copolymer used for the OV coating was carried out by a modified version of the Reversible Addition-Fragmentation chain Transfer technique (RAFT), which allows for reducing the time required for each block synthesis [35,36] and avoids intermediate purification steps (Scheme 1).

O-(β-D-galactopyranosylethyl) acrylamide (Gal, compound **6**) was initially polymerized to generate the first block (Gal*_n_*_,_ compound **8**) using 1:32 RAFT agent:Gal molar ratio. Under the operative conditions, 100% monomer conversion was achieved in 2 h as checked by ^1^H NMR analysis (Figure S11). The obtained macro-Chain Transfer Agent (macro-CTA) Gal_32_ was then chain-extended by polymerization of the positively charged monomer agmatine-acrylamide (Agm, compound **7**) using 1:32 macro-CTA Gal_32_:Agm molar ratio to obtain a diblock copolymer with a similar number of monomer units per block. The polymerization yielded 90% monomer conversion, which corresponded to Gal_32_-*b*-Agm_29_ (**9**). After purification by dialysis to remove the excess of unreacted Agm, the isolated diblock copolymer was characterized by GPC and ^1^H NMR. The GPC chromatogram indicated that the copolymer had a low polydispersity index (PDI 1.4) and an M_n_ of 21.9 kDa, which is higher than the theoretical value (M_n,th_ = 14.5 kDa). However, it is well documented that the estimation of molecular weight of cationic polymers can be subjected to undesired phenomena, such as ion and hydrophobic interactions or ion exclusion, which affect the analytical accuracy [47,48]. The ^1^H NMR spectrum (Figures S11 and S12) and the GPC analysis (Figure S13) confirmed the copolymer Gal:Agm molar ratio and the successful purification from unreacted species.

### 3.2. Oncolytic Adenoviruses Coating with Gal_32_-b-Agm_29_

**Figure 1:**
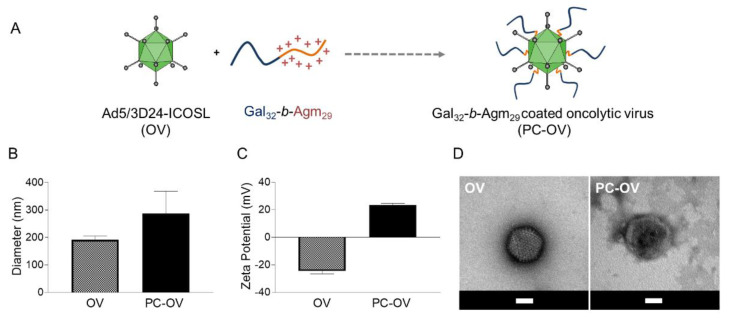
Surface modified oncolytic adenovirus (OV) as targeting strategies. (**A**) Schematic representation of the strategy to generate the OVs surface with ASPGR-targeting macromolecules. A cation block was added to the polymer to confer a positive charge that favors the interaction with the negatively charged capsid of the virus, allowing the formation of a complex (PC-OV). (**B**,**C**) Viral particles were coated with an excess of Gal_32_-b-Agm_29_ polymer chains. After the reaction, zeta potential (**B**) and particle size (**C**) were measured. Data are reported as mean ±SD. (**D**) Electron microscopy pictures of naked virus and PC-OV (scale bar 50 nm).

The Gal_32_-*b*-Agm_29_/OV complexation was achieved by simple incubation of OV with diblock copolymer to yield the full coating of OVs by coulombic interactions (Figure 1A) [49]. The polymer-coated virus (PC-OV) was characterized by dynamic light scattering (DLS), zeta potential, and transmission electron microscopy (TEM) to assess the effect of the galactosylated polymer on the diameter, surface charge, and morphology of the viral particles (Figure 1B–D).

The DLS results reported in Figure 1B and zeta-potential results reported in Figure 1C confirmed the OV coating. Indeed, the polymer coating induced a shift in both OV hydrodynamic size from 191.9 ± 13.6 nm with a PDI of 0.304 ± 0.036 for the naked OV to 287 ± 81 nm with a PDI of 0.428 ± 0.036 for the PC-OV, and zeta potential from −24.7 ± 1.78 mV for the naked OV to 23.3 ± 1.29 mV for the PC-OV. The polymer adhesion to the viral particle surface also induced a modification of the virus aspect. The TEM images reported in Figure 1D show the typical icosahedral symmetrical structure for the naked OV with an average particle diameter of 105 nm. After coating with Gal_32_-*b*-Agm_29_, the PC-OV size was about 140 nm, while the icosahedral aspect was hidden by the polymer shell, as already reported for polymer-coated viruses [50]. The discrepancy between the sizes obtained by DLS and TEM can be ascribed to the aggregation phenomena of a single virus during DLS analysis or to the drying process required for the TEM sample, as shown already by Nikitin et al. [51].

### 3.3. Oncolytic Viruses Complexed with Galactosylated Polymers Exhibit Enhanced In Vitro Efficacy in HepG2 Hepatocarcinoma Cell Line

Cytotoxicity studies were carried out to gain insights into the potential therapeutic value of PC-OV and its antitumor activity using the HepG2 human hepatoma cell line, which expresses high levels of ASGPRs that bind galactosyl moieties [52], and A549 adenocarcinomic human alveolar basal epithelial cells lacking ASGPR expression, which were used as negative control [53].

**Figure 2:**
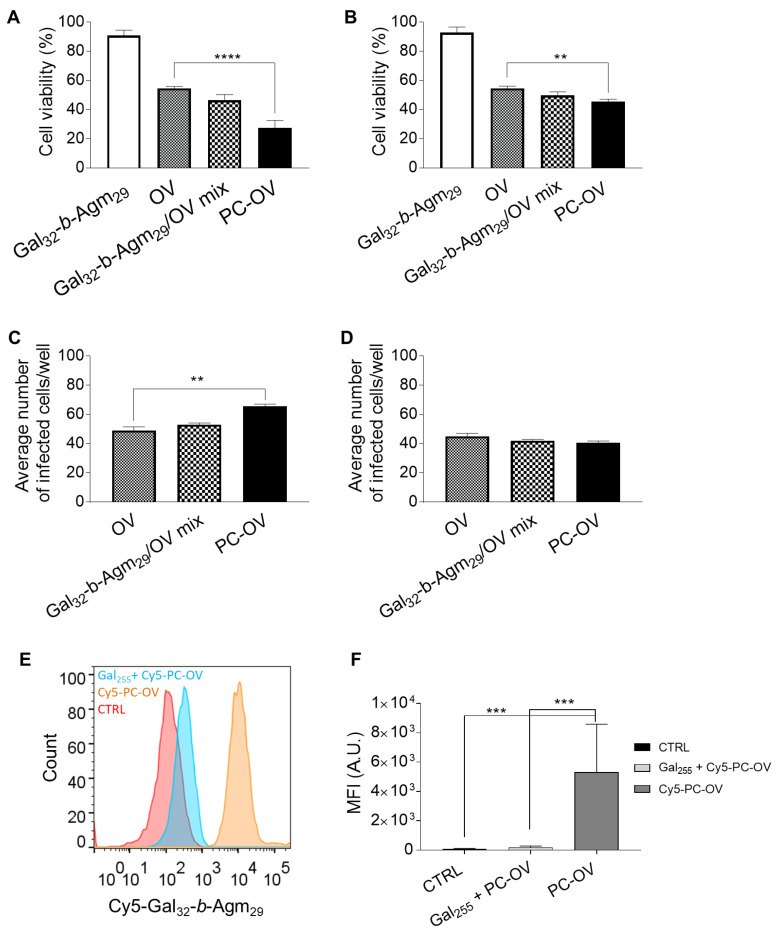
Cell viability, infection titer, and competition studies. (**A**,**B**) In vitro cytotoxicity of naked OV, Gal_32_-*b*-Agm_29_ alone, Gal_32_-*b*-Agm_29_/OV mix, and PC-OV was measured by a MTS cell-viability assay at day 3 post-treatment on (**A**) HepG2 and (**B**) A549 cells. Control untreated cells were used as reference (100% cell viability); (**C**,**D**) the infectivity of naked OV, Gal_32_-*b*-Agm_29_/OV mix, and PC-OV was assessed by ICC assay in HepG2 (**C**) and A459 (**D**) cell lines, based on visual quantification of infected cells after the staining of virus hexon protein. For every five replicates, five images of non-overlapping fields were acquired using an AMG EVO XL microscope. For infectivity comparison, the data are presented as the average number of infected cells per well. Error bars, mean ±SEM: ** *p* < 0.01; **** *p* < 0.0001. (**E**,**F**) Flow cytometry histogram showing cellular uptake of Cy5-Gal_32_-*b*-Agm_29_ after incubation with Cy5-Gal_32_-*b*-Agm_29_/OV complex (Cy5-PC-OV) along with control (CTRL) cells (**E**) and comparative median fluorescence intensity (MFI) (**F**) of control cells (CTRL), cells pre-incubated with Gal_255_ polymer followed by incubation with Cy5-PC-OV (Gal_255_ + Cy5-PC-OV), and cells incubated with Cy5-PC-OV only. Error bars, mean ± SD; *** *p* < 0.001.

Cells were incubated with OV, PC-OV, Gal_32_-*b*-Agm_29_ alone, and a mixture of Gal_32_-*b*-Agm_29_ and OV (Gal_32_-*b*-Agm_29_/OV mix) separately added to the wells. To comparatively investigate the cytotoxicity and infectivity of samples, cells were incubated with equivalent OV and/or Gal_32_-*b*-Agm_29_ concentrations (Figure 2A–D).

Figure 2A,B shows that HepG2 and A549 cell viability was above 90% when incubated with Gal_32_-*b*-Agm_29_ alone for 72 h, indicating that this copolymer is per se biocompatible, which is a prerequisite for its pharmaceutical use. On the contrary, all samples containing OV were found to induce cell death, although to a different extent.

The cytotoxicity observed with naked OV was due to the cell internalization of Ad5/3-D24-ICOSL through DSG2 and CAR receptors [42], which were preliminarily found to be expressed by both HepG2 and A549 cells (Figure S14). Despite the lower expression of DSG2 and CAR receptors in A549 cells as compared to HepG2 cells, similar cytotoxicity was observed in both cell lines. This is probably due to the OV internalization by A549 cells through other receptors, including CD46 [54] or GD1a glycan [55], known to be overexpressed to a higher level by this cell line in comparison to HepG2 cells as reported by Gao et al. [56].

Gal_32_-*b*-Agm_29_/OV mix displayed slightly higher cytotoxicity than naked OV in both cell lines, suggesting that the free copolymer added to the cells may have some limited effect in promoting the OV cell penetration. It should be noted that macromolecules containing positive charges, such as guanidyl groups, favor the passive cell internalization of small molecules and colloidal systems [57].

Conversely, PC-OV exhibited higher cytotoxicity towards HepG2 than A549 cells. More importantly, in HepG2, PC-OV displayed enhanced cytotoxicity as compared to naked OV and Gal_32_-*b*-Agm_29_/OV mix (about 30% and 20% higher, respectively), while in A549 cells, the differences between the tested formulations were negligible (PC-OV toxicity about 9% and 4% higher as compared to OV and Gal_32_-*b*-Agm_29_/OV mix, respectively). The different results obtained with the two cell lines were in agreement with the high HepG2 expression of ASGPR, while A549 did not express these galactose-specific receptors, confirming that the cell uptake of PC-OV is mediated by the galactosyl coating. Cytotoxicity was additionally evaluated in the presence of increasing serum concentration (2–20% FBS) in HepG2 cells (Figure S15), showing that the presence of serum protein has a limited impact on infectivity and therefore killing for all the tested formulations. In particular, PC-OV exhibited a negligible variation (5.6% increase in cell viability from 2 to 20% FBS) ascribable to the stealth properties provided by the galactosyl shell.

The infectivity of the different formulations (OV, Gal_32_-*b*-Agm_29_/OV mix, PC-OV) was further investigated by an immunocytochemistry assay (ICC). Figure 2C,D shows that in HepG2 cells, the infectious titer was significantly higher in the case of PC-OV than for naked OV and Gal_32_-*b*-Agm_29_/OV mix, while in A549 cells, where ASGPRs are not expressed, all samples displayed comparable infectious titer. These results support our hypothesis that the enhanced internalization of PC-OV in comparison to OV and Gal_32_-*b*-Agm_29_/OV mix is mediated by the galactosyl block of the copolymer on the viral surface which is responsible for the interaction with ASGPRs. This was further confirmed by the competition study (Figure 2E,F), where HepG2 cells were pre-incubated with 250 μg/mL Gal_255_ polymer for one hour before the addition of Cy5-Gal_32_-*b*-Agm_29_/OV complex. The presence of an excess of galactose binding units displayed by Gal_255_ polymer and free to interact with the ASGPR almost completely abolished the Cy5-Gal_32_-*b*-Agm_29_/OV complex internalization by HepG2 cells.

**Figure 3:**
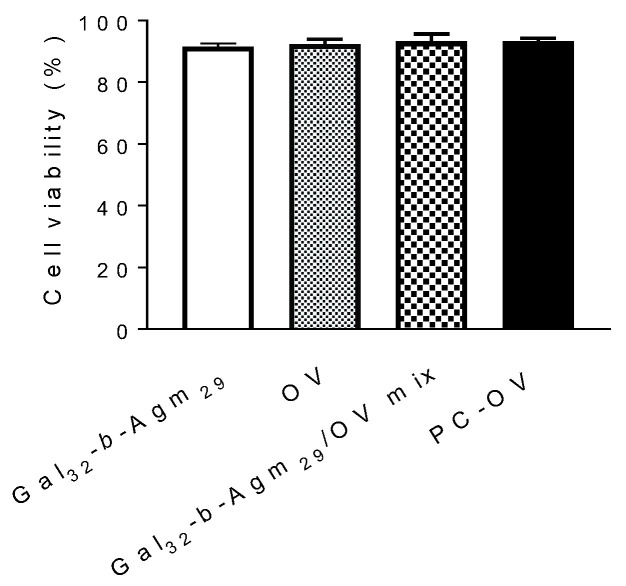
Cell viability on human juvenile fibroblasts. Cell viability was performed by MTS assay on juvenile fibroblasts (incubation time 96 h). The absorbance was measured with a 96-wells plate spectrophotometer Varioskan Flash Multimode Reader set at the wavelength of 490 nm.

The overall tumor cell selectivity of PC-OV was examined by using nontumor cells juvenile fibroblasts. The results reported in Figure 3 show that none of the tested samples displayed significant cytotoxicity (cell availability > 90%), confirming the excellent biocompatibility of the copolymer and the pharmacological selectivity of OV for tumor cells.

The number of apoptotic and necrotic cells was assessed to investigate in depth the effect of the virus coating on the pharmacological performance of OV.

**Figure 4:**
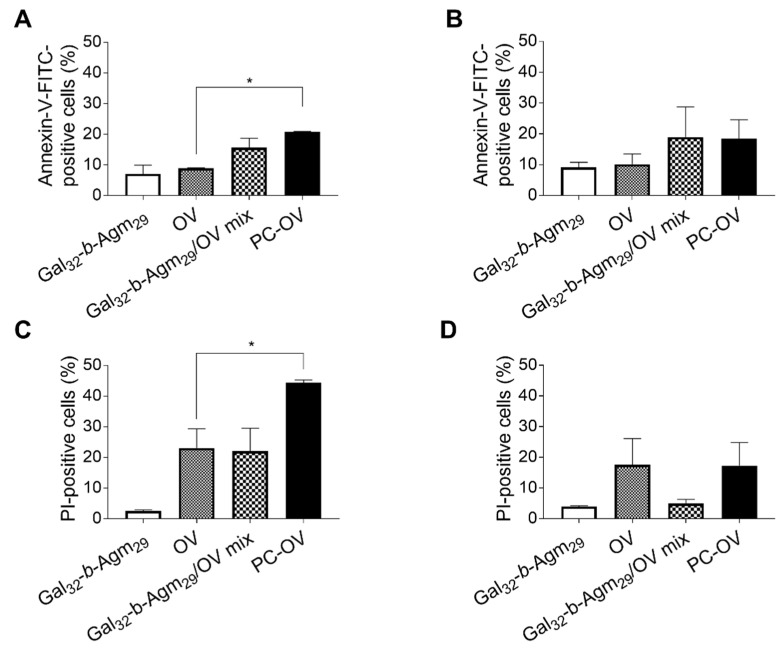
Induction of apoptotic and necrotic cell death. Early apoptotic (**A**,**B**) and late apoptotic/necrotic (**C**,**D**) cell death in HepG2 (**A**,**C**) and A549 (**B**,**D**) cells at 48 h post-treatment. FITC-labeled Annexin-V and PI were used to detect the early apoptotic cell and the necrotic or late apoptotic cells, respectively. Representative dot plots and histogram analyses are shown in Figures S16 and S17. Error bars, mean ± SEM; * *p* < 0.05.

Figure 4A,C show that HepG2 incubation with PC-OV resulted in an increased number of apoptotic and necrotic cells (>40%), as compared to the other samples. On the contrary, Figure 4B,D show that the number of apoptotic and necrotic A549 cells was generally low (below 20%) in all treatments.

Taken together, these results suggest the potentiality of retargeted oncolytic adenoviruses for the treatment of HCC. Indeed, in HepG2, PC-OV displayed enhanced infectivity and improved anticancer properties over naked OV, suggesting that coating with targeting polymer can be properly exploited to enhance the efficacy of oncolytic virotherapy in cancer treatment.

### 3.4. Oncolytic Viruses Coated with Galactosylated Polymers Induce Immunogenic Cell Death (ICD)

Cancer development and spread is a process of biological evolution that consists of genetic changes and various adaptations. Different mutations confer a specific benefit depending on the changes in the tumor microenvironment. It has been reported that, in general, the success of the anticancer pharmacological therapies relies on the induction of immunogenic cell death (ICD) and the development of antitumor immune responses [58]. Cells undergo induction of ICD due to endoplasmic reticulum (ER) stress and reactive oxygen species (ROS) production as components for the exposure of different damage-associated molecular pathways (DAMPs) [59]. After the induction of ICD, chronic exposure of DAMPs on cancer cells attracts receptors and ligands on dendritic cells (DCs) and activates DCs to transition to a mature phenotype, which consequently results in stimulating specific T cell responses and subsequent killing of more cancer cells.

Similar to a few therapeutic agents, OVs are potent ICD activators enhancing the anticancer immune response [60] that contributes to antitumor efficacy. Furthermore, oncolytic adenoviruses can be genetically modified to complement mutations within the signaling pathways of ICD, resulting in clinical benefits. In particular, OVs can be constructed to produce caspase-8 in order to sensitize ICD resistant cancer types [59]. ICD relies on DAMPs and the signaling pathways that mediate the overexpression of ICD biomarkers, such as calreticulin (CRT), in the outer plasma membrane, followed by the extracellular release of high-mobility group box 1 protein (HMGB1) and adenosine triphosphate (ATP) [61,62]. Therefore, to evaluate whether a program of immunogenic cell death is triggered by OV treatments, the presence of HMGB1 and adenosine ATP in the supernatants is assessed [58,63].

**Figure 5:**
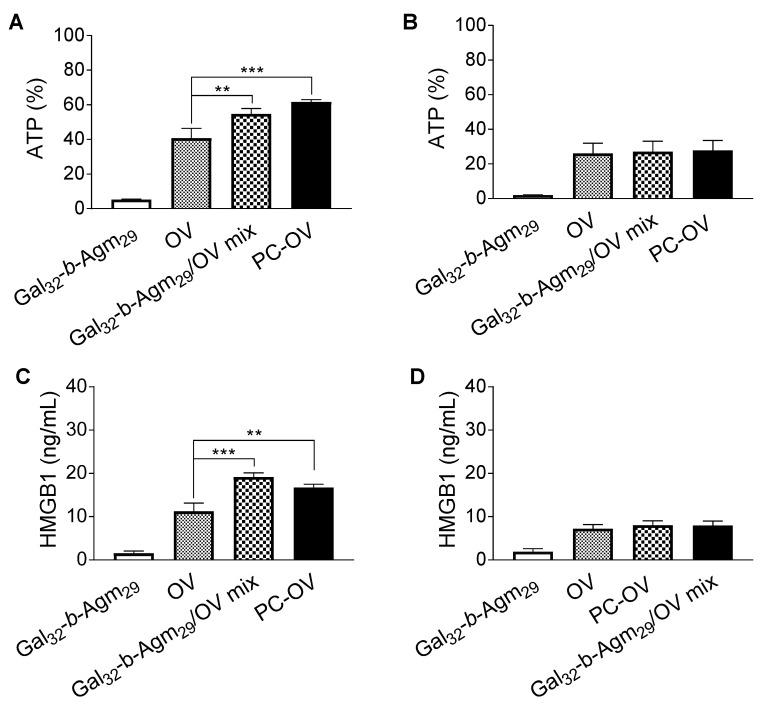
In vitro immunogenic tumor cell death. Extracellular ATP (**A**,**B**) in the supernatant of HepG2 (**A**) and A549 (**B**) cells after 48 h of treatment. Extracellular HMGB1 (**C**,**D**) in the supernatant of HepG2 (**C**) and A549 (**D**) cells after 72 h of treatment. Error bars, mean ± SEM: ** *p* < 0.01, *** *p* < 0.001.

Figure 5 reports the levels of ICD marker released by HepG2 and A549 cells treated with the same OV and/or Gal_32_-*b*-Agm_29_ concentrations of naked OV, PC-OV, Gal_32_-*b*-Agm_29_ alone, and Gal_32_-*b*-Agm_29_/OV mix.

The results reported in Figure 5 show that Gal_32_-*b*-Agm_29_ alone did not elicit significant expression of the ICD markers, which confirmed the excellent biocompatibility of this copolymer, while all samples containing OV (naked OV, PC-OV, and Gal_32_-*b*-Agm_29_/OV mix) were found to elicit relevant expression of ICD markers.

The higher release of the ICD marker in HepG2 cells as compared to A549 cells observed with all samples containing OV can be explained by the different ICD sensitivity of the two cell lines. Indeed, although both lung adenocarcinoma (A549) and HCC (HepG2) are immunogenic tumors [64], HepG2 might be more sensitive than A549 to changes induced by the virus infection resulting in the higher release of ICD markers, similarly to what is reported in the literature with other cell lines [61].

In the case of HepG2 cells, the highest immunogenic cell death was observed with PC-OV, which yielded higher levels of ATP (60%) and HMGB1 (20%) as compared to A549 cells (28% ATP and 8% HMGB1). Furthermore, in A549 no significant differences were observed among the various treatments. These results suggest that the presence of galactosylated polymer improves the OV delivery into HCC cells, thus boosting the infectivity and enhancing the in vitro therapeutic efficacy through the increased release of ATP and HMGB1.

## 4. Conclusions

In this study, we supported the use of cationic galactosylated polymers as carriers for the systemic delivery of oncolytic adenoviruses in the hepatocellular carcinoma cell line. Our results demonstrated the ability of OVs complexed with Gal_32_-*b*-Agm_29_ to improve the virus cell entry, thus enhancing viral replication that ultimately leads to the cancer cell lyses and higher immunogenic cell death induction program in the hepatocellular carcinoma cell line.

Altogether, here, we have proved that the use of galactosylated polymers for both coating and targeting is a safe and efficacious therapeutic strategy that minimizes side effects. Obtained results provide a rationale for future studies utilizing oncolytic viruses complexed with polymers towards specific and more effective targeting of HCC.

## Data Availability

Not applicable.

## Supplementary Materials

The following are available online at https://www.mdpi.com/article/10.3390/pharmaceutics13070949/s1, Scheme S1: Synthesis of DMTMM; Scheme S2: Synthesis of 4-cyano-4-(ethylsulfanylthiocarbonylsulfanyl)pentanoic acid; Scheme S3. Synthesis of D-galactopyranosyloxyethyl acrylamide; Scheme S4: Synthesis of agmatine acrylamide; Scheme S5: Synthesis of Cy5-Gal_32_-*b*-Agm_29_ by coupling reaction; Figure S1: ^1^H NMR of CDMT in CDCl_3_; Figure S2: ^13^C NMR of CDMT in CDCl_3_; Figure S3: ^1^H NMR of DMTMM in MeOH-*_d4_*; Figure S4: ^13^C NMR of DMTMM in MeOH-*_d4_*; Figure S5: ^1^H NMR in CDCl_3_ of purified 4-cyano-4(ethylsulfanylthiocarbonylsulfanyl)pentanoic acid; Figure S6: ^13^C NMR in CDCl_3_ of purified 4-cyano-4(ethylsulfanylthiocarbonylsulfanyl)pentanoic acid; Figure S7: ^1^H NMR of β-D-galactopyranosyloxyethyl acrylamide in DMSO-*_d6_* after purification; Figure S8: ^13^C NMR of β-D-galactopyranosyloxyethyl acrylamide in DMSO-*_d6_* after purification; Figure S9: ^1^H NMR of agmatine acrylamide in D_2_O after purification; Figure S10: ^13^C NMR of agmatine acrylamide in D_2_O after purification; Figure S11: ^1^H NMR in DMSO-*_d6_* of macro-CTA Gal_32_; Figure S12: ^1^H NMR in DMSO-*_d6_* of Gal_32_-*b*-Agm_29_ block copolymer after purification by dialysis; Figure S13: Gel permeation chromatography (GPC) profile of Gal_32_-*b*-Agm_29_ (**9**) block copolymer after purification by dialysis and freeze-drying; Figure S14: Positive cells for CAR and DSG2 measured via flow cytometry by specific antibody staining; Figure S15: In vitro cytotoxicity on HepG2 cells at increasing concentration of serum; Figure S16: Dot plot with percentage of Annexin V/PI positive HepG2 and A549 cells; Figure S17: Representative flow cytometry analysis of Annexin V/ PI assay performed on HepG2 and A549 cells.
