# Supplementary material for: Polymer Coated Oncolytic Adenovirus to Selectively Target Hepatocellular Carcinoma Cells
Source: Pharmaceutics. 2021 Jun 24;13(7):949. doi: 10.3390/pharmaceutics13070949 (PMC8309094; doi:10.3390/pharmaceutics13070949)
Supplement: Supplementary file 1 [file pharmaceutics-13-00949-s001.zip › pharmaceutics-1237595-SI.pdf]

pharmaceutics

Supplementary Materials: Polymer Coated Oncolytic Adenovirus to Selectively Target Hepatocellular Carcinoma Cells

Mariangela Garofalo, Federica Bellato, Salvatore Magliocca, Alessio Malfanti, Lukasz Kuryk, Beate Rinner, Samuele Negro, Stefano Salmaso, Paolo Caliceti, Francesca Mastrotto

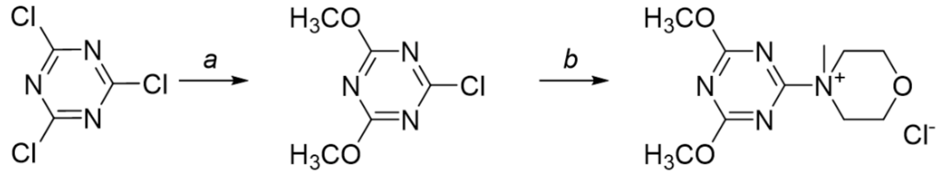

(1) (2)

Scheme S1. Synthesis of CDMT (1) intermediate and DMTMM (2). *Reagent and conditions*: **(a)** MeOH, milliQ water, Na_2CO_3, from 0 to 35 °C, 12 h; **(b)** *N*-methylmorpholine, THF, room temperature, 30 min.

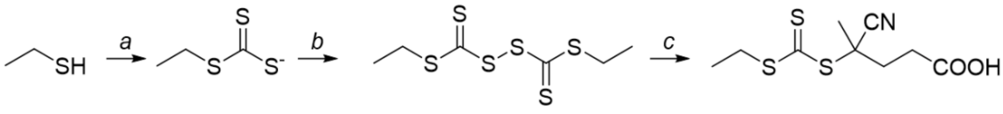

(3) (4)

Scheme S2. Synthesis of bis(ethylsulfanylthiocarbonyl)disulfide (3) intermediate and 4-cyano-4-(ethylsulfanylthiocarbonylsulfanyl)pentanoic acid (4). *Reagents and conditions*: **(a)** CS_2, NaOH, water:acetone 4:1, 0 °C, 1 h; **(b)** $K_3[Fe(CN)_6]$; **(c)** 4,4′-azobis(cyanopentanoic acid), EtOH, 60 °C, 16 h.

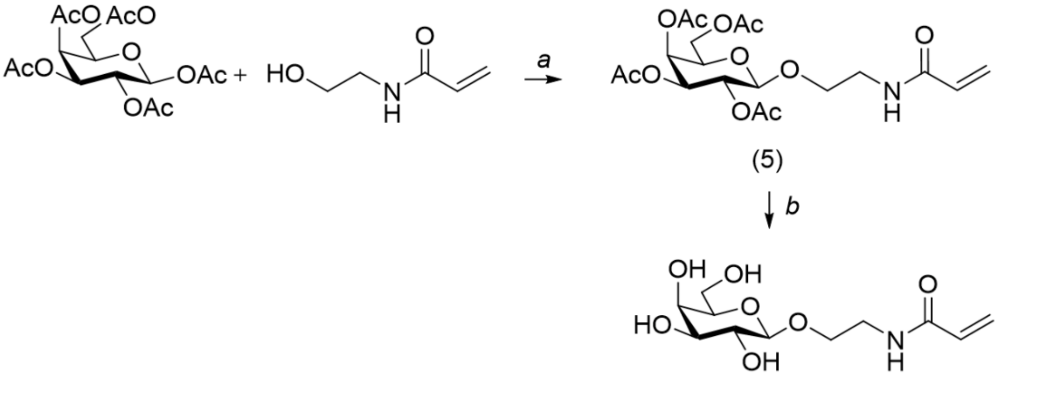

(6)

Scheme S3. Synthesis of 2,3,4,6-O-tetraacetyl-D-galactopyranosyloxyethyl acrylamide (5) and its deprotection to yield D-galactopyranosyloxyethyl acrylamide (6). *Reagents and conditions*: **(i)** BF_3Et_2O, CH_2Cl_2, 0°C; **(ii)** KOH, CH_3OH.

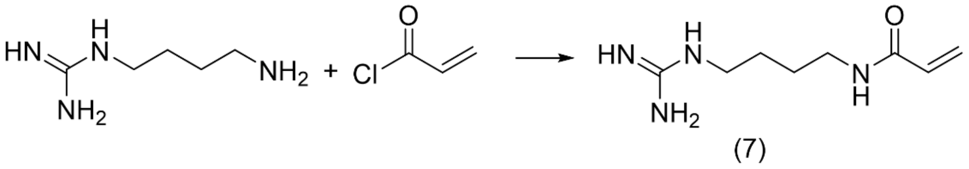

Scheme S4. Synthesis of agmatine acrylamide (7). *Reagents and conditions*: K_2CO_3, NaOH, 0 °C, 16 h.

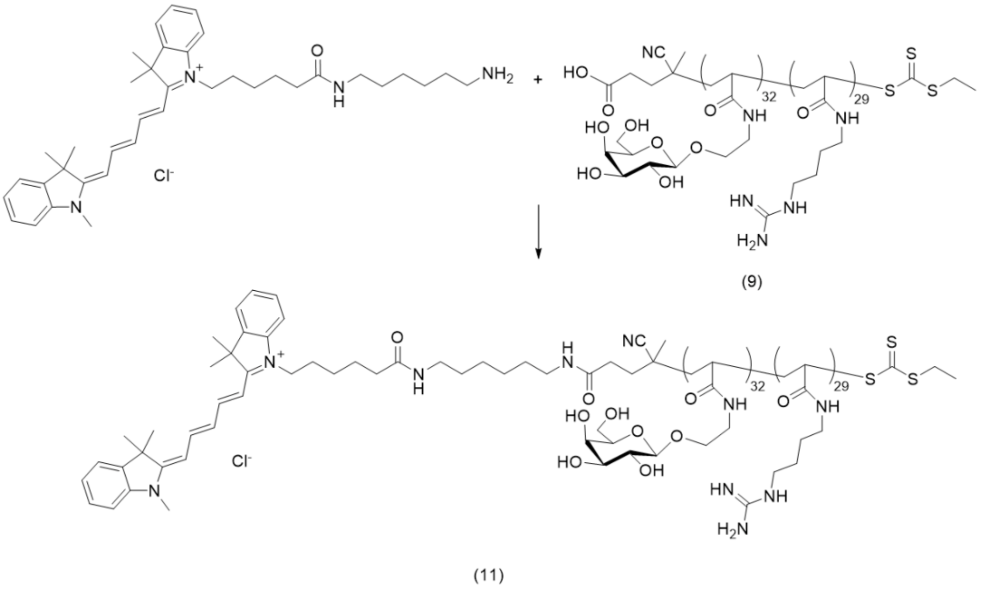

Scheme S5. Synthesis of Cy5-Gal_{32}-*b*-Agm_{29} (11) by coupling reaction of Cy5-NH_2 with Gal_{32}-*b*-Agm_{29} copolymer. *Reagent and conditions*: DMTMM, 50 mM MES pH 6.8, room temperature, 16 h.

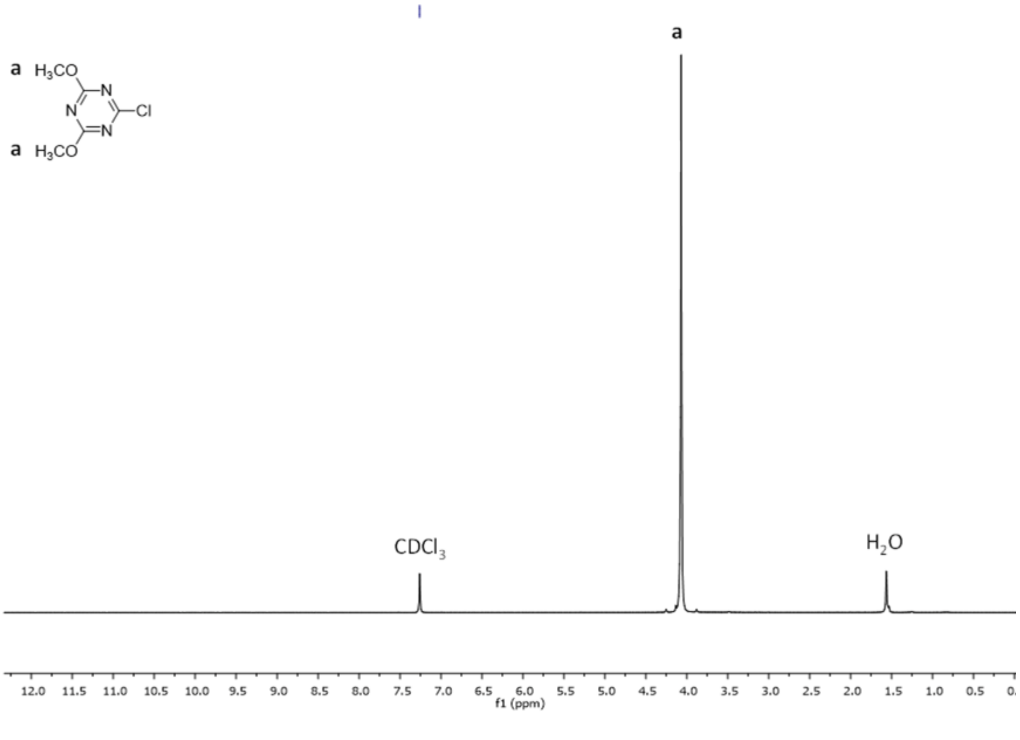

Figure S1. ^{1}H NMR of CDMT (1) in $CDCl_3$.

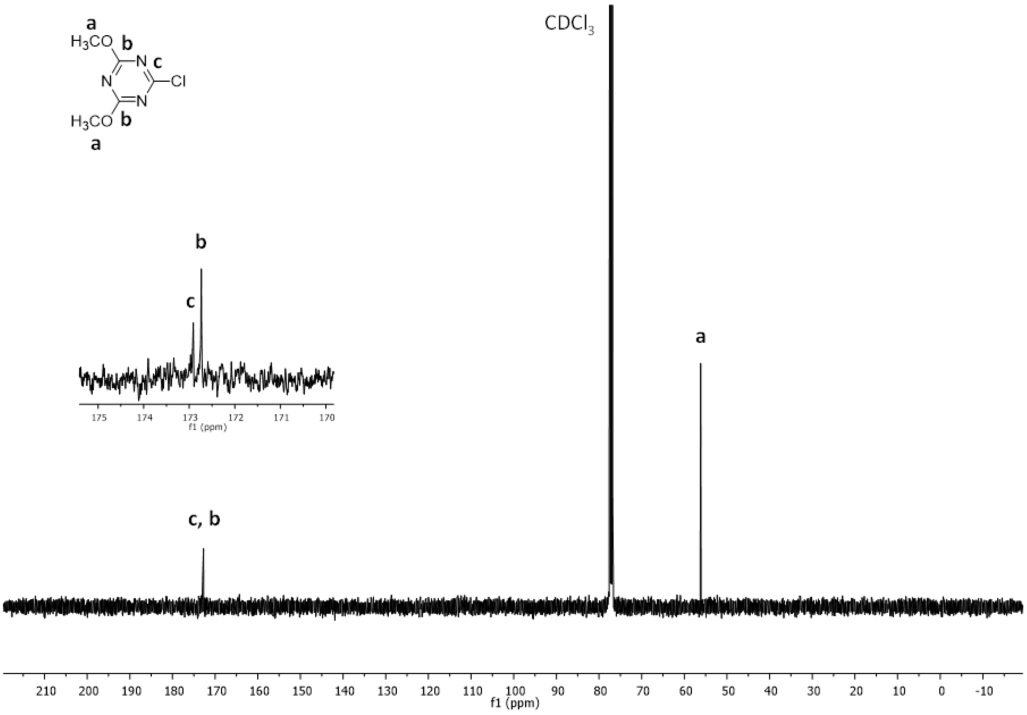

Figure S2. ^{13}C NMR of CDMT (2) in $CDCl_3$.

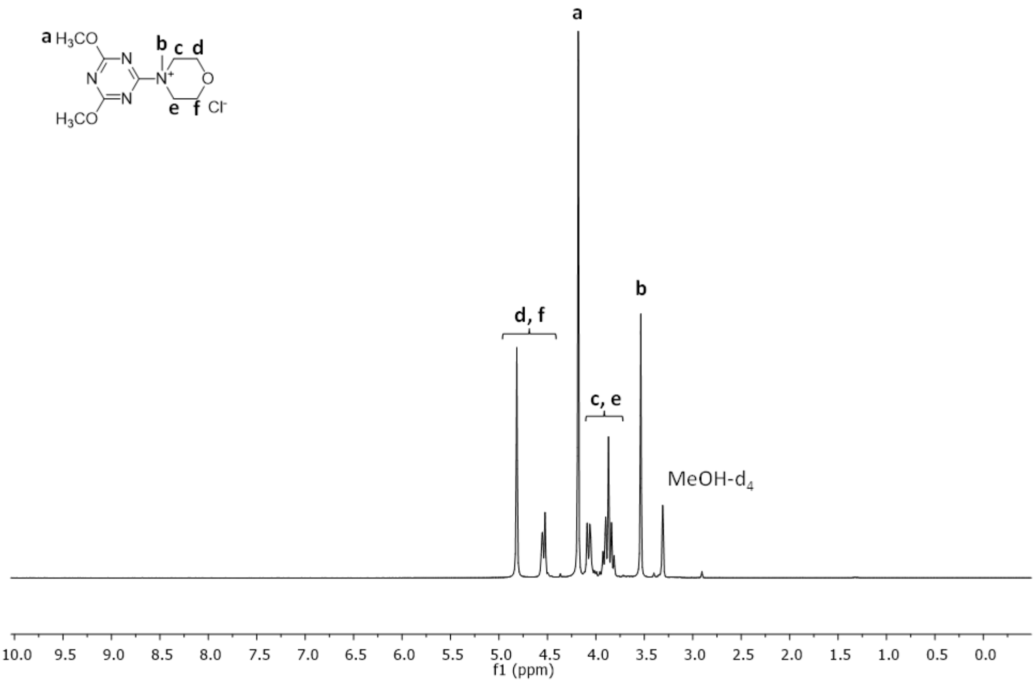

Figure S3. ^{1}H NMR of DMTMM (2) in MeOH-d_4.

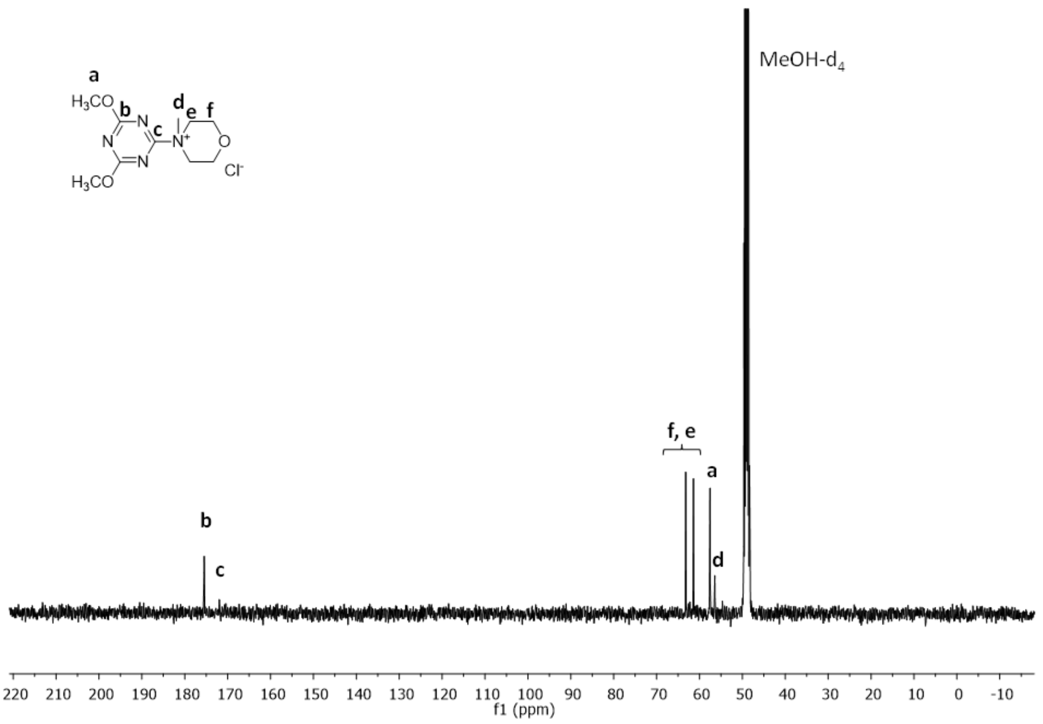

Figure S4. ^{13}C NMR of DMTMM (2) in MeOH-*d*4.

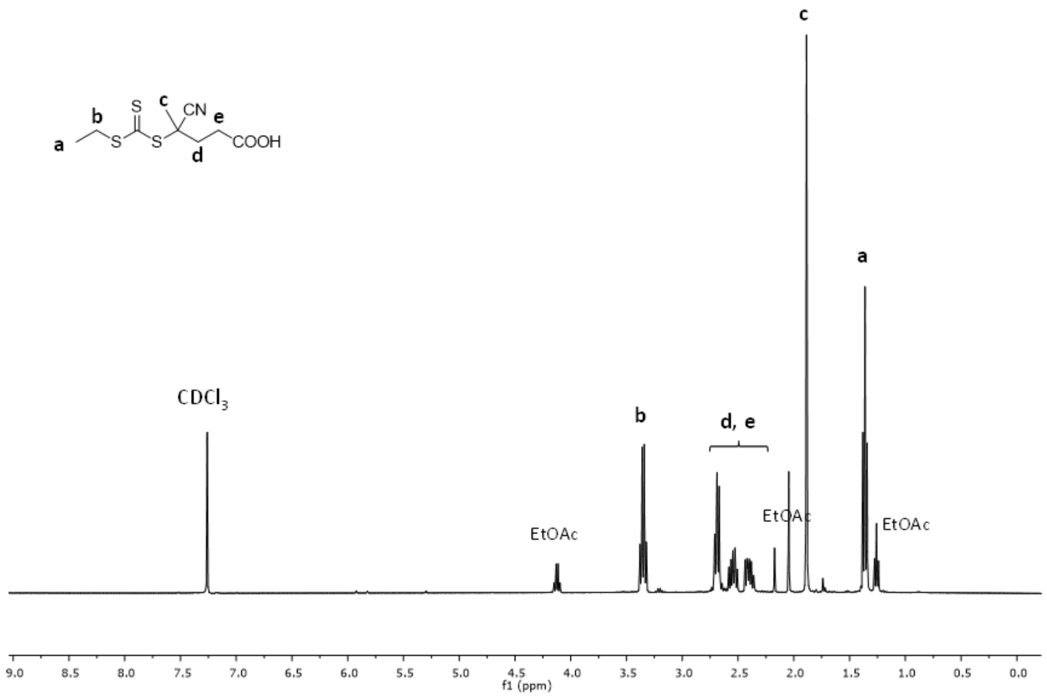

Figure S5. ^{1}H NMR in $CDCl_3$ of purified 4-cyano-4(ethylsulfanylthiocarbonylsulfanyl)pentanoic acid (4).

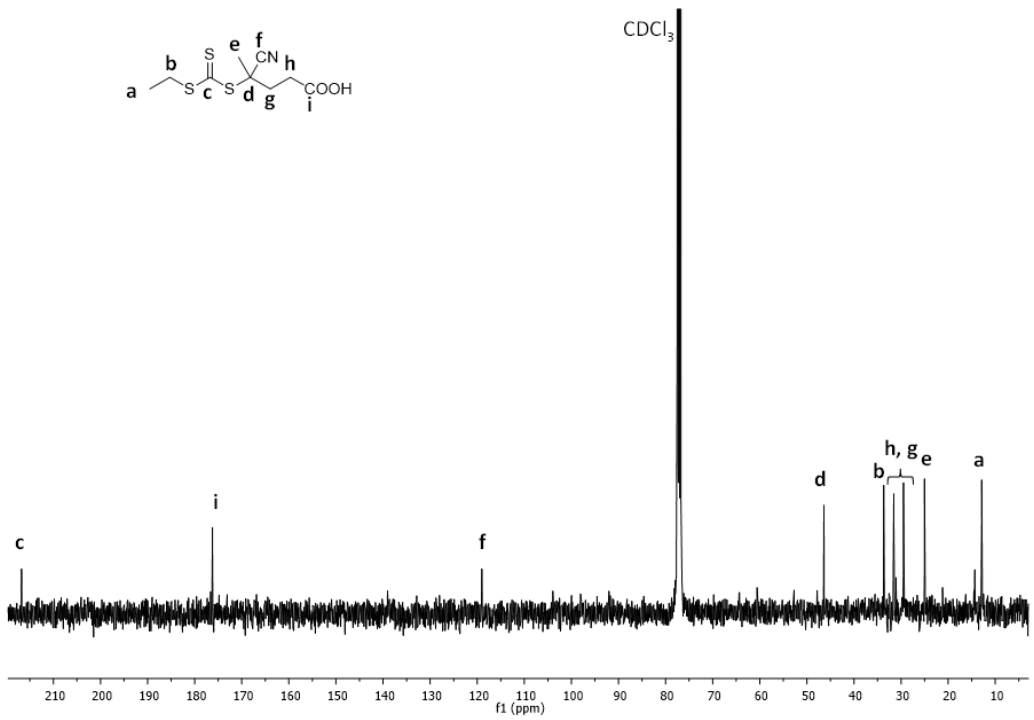

Figure S6. ^{13}C NMR in $CDCl_3$ of purified 4-cyano-4(ethylsulfanylthiocarbonylsulfanyl)pentanoic acid (4).*Signals at 14.34, 21.19, 60.58 ppm were due do EtOAc traces.

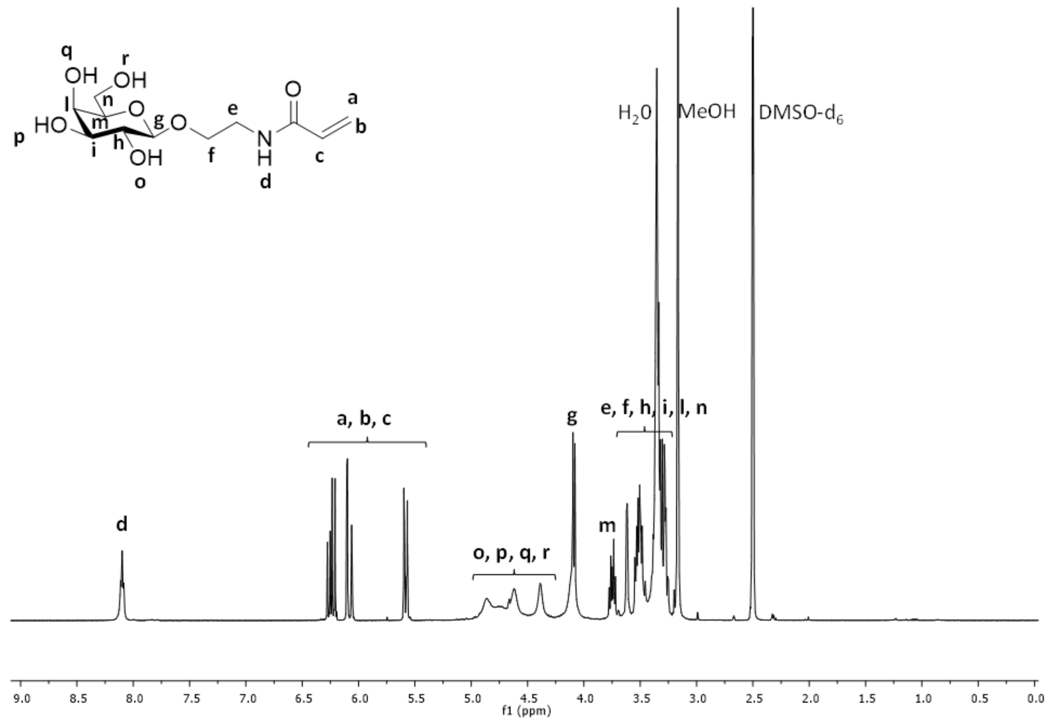

Figure S7. ^{1}H NMR of β–D-galactopyranosyloxyethyl acrylamide (6) in DMSO-d_6 after purification.

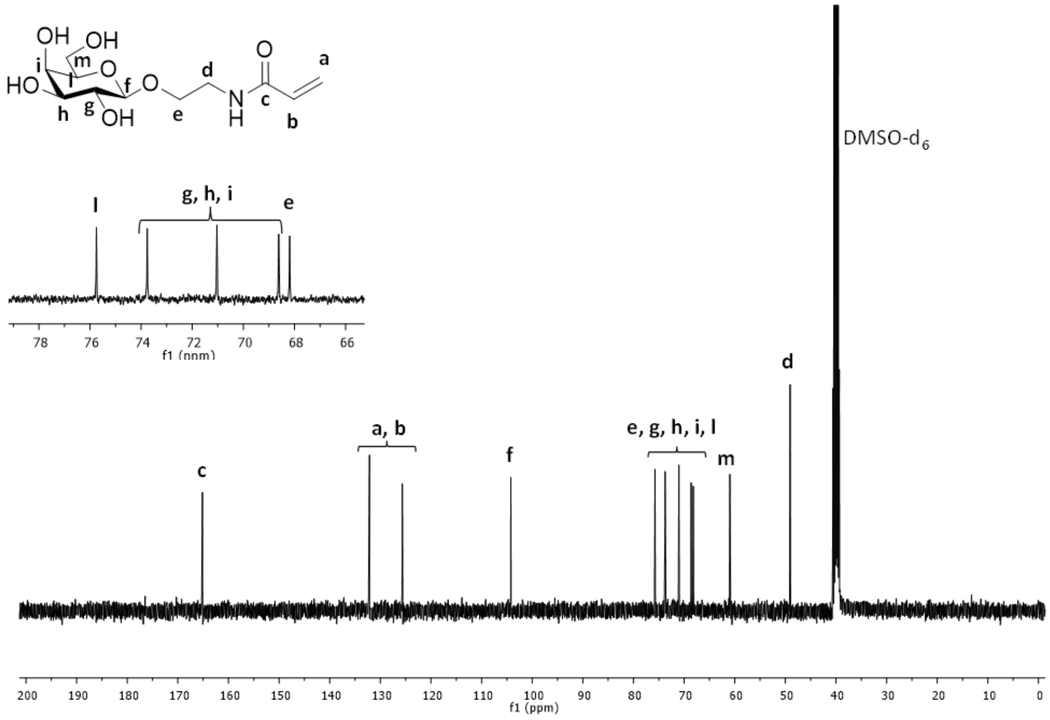

Figure S8. ^{13}C NMR of β–D-galactopyranosyloxyethyl acrylamide (6) in DMSO-d_6 after purification.

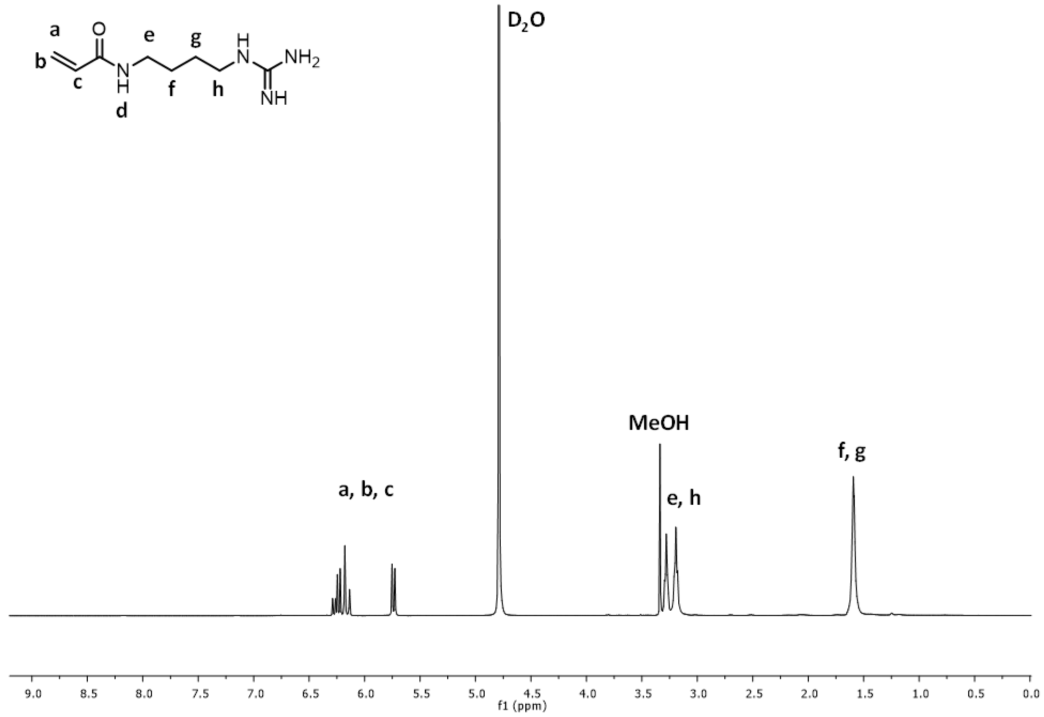

Figure S9. ^{1}H NMR of agmatine acrylamide (7) in D_2O after purification.

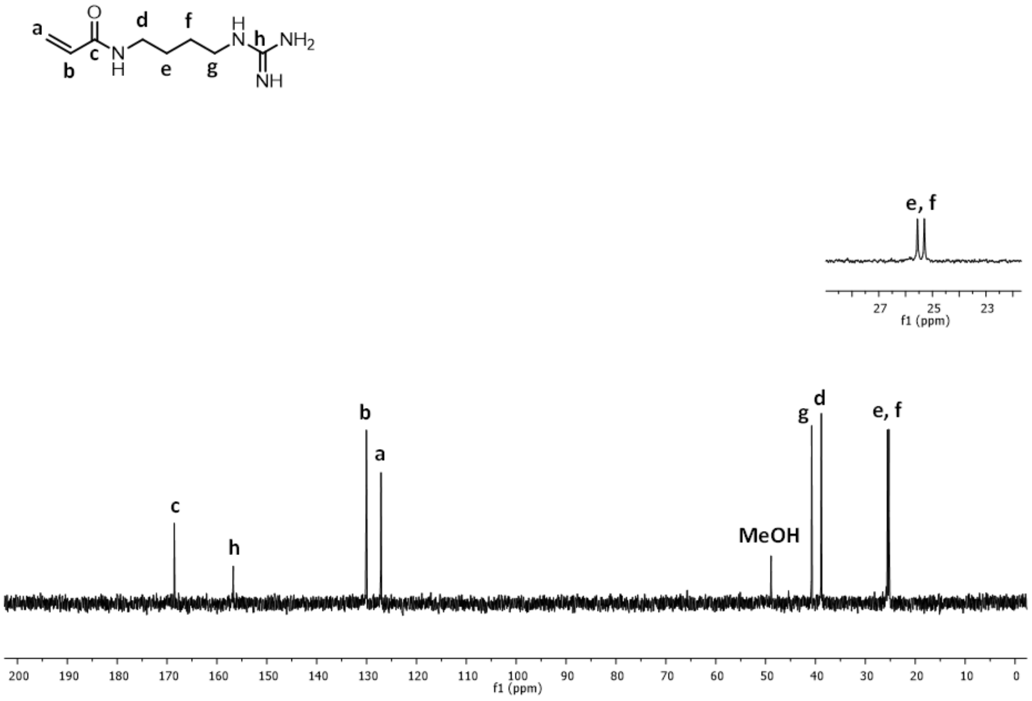

Figure S10. ^{13}C NMR of agmatine acrylamide (7) in D_2O after purification.

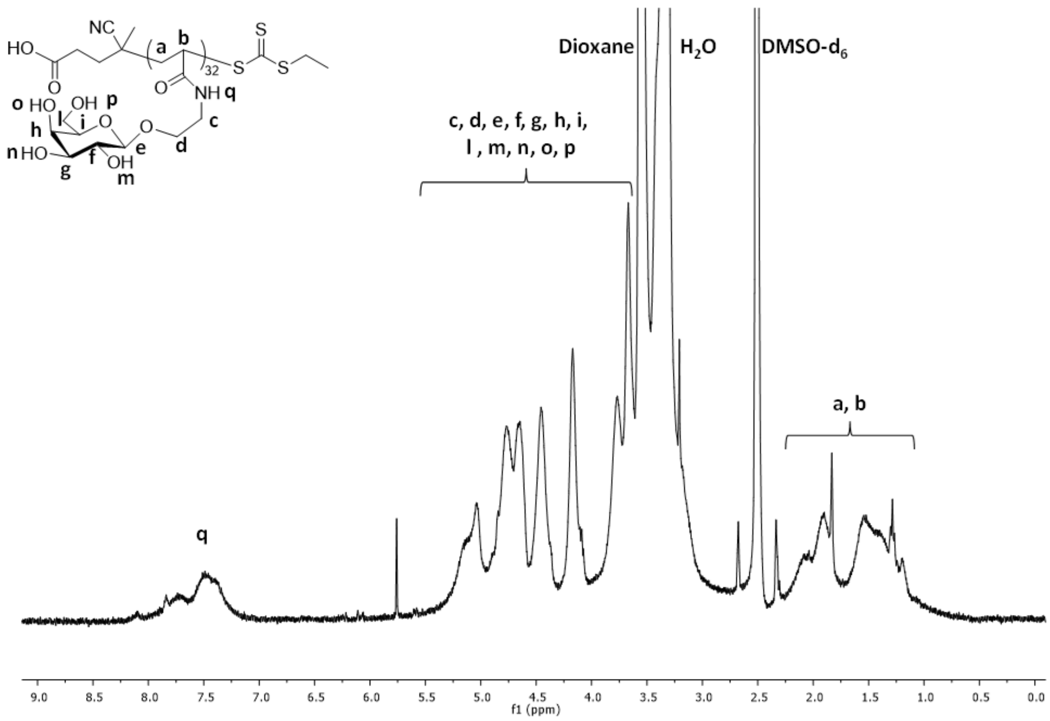

Figure S11. ^{1}H NMR in DMSO-d_6 of macro-CTA Gal_{32} (8) performed on an aliquot of the polymerisation mixture withdrawn at the end of the first polymerisation step. The intermediate was used for the next polymerisation step without isolation or purification.

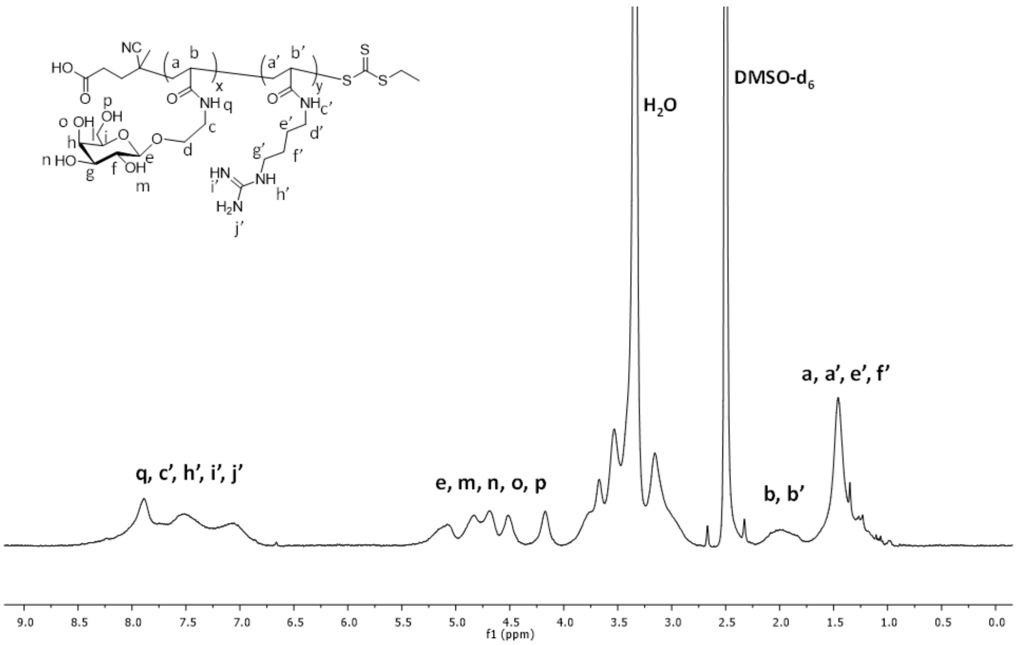

Figure S12. ^{1}H NMR in DMSO-*d*6 of Gal32-*b*-Agm29 (9) block copolymer after purification by dialysis.

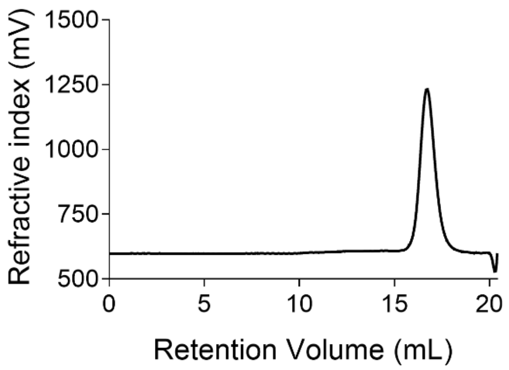

Figure S13. Gel permeation chromatography (GPC) profile of Gal32-*b*-Agm29 (9) block copolymer after purification by dialysis and freeze-drying.

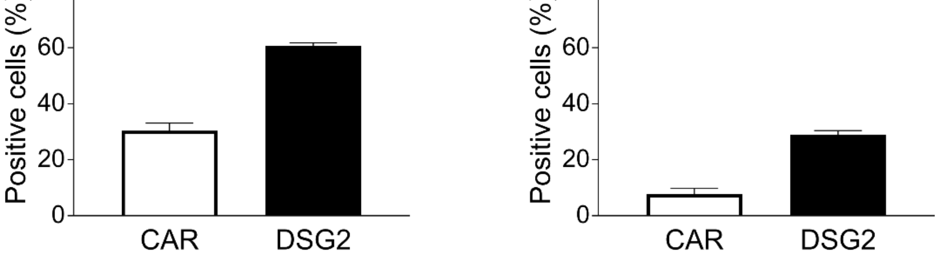

Figure S14. HepG2 (left panels) and A549 (right panels): positive cells for CAR and DSG2 were measured via flow cytometry by specific antibody staining.

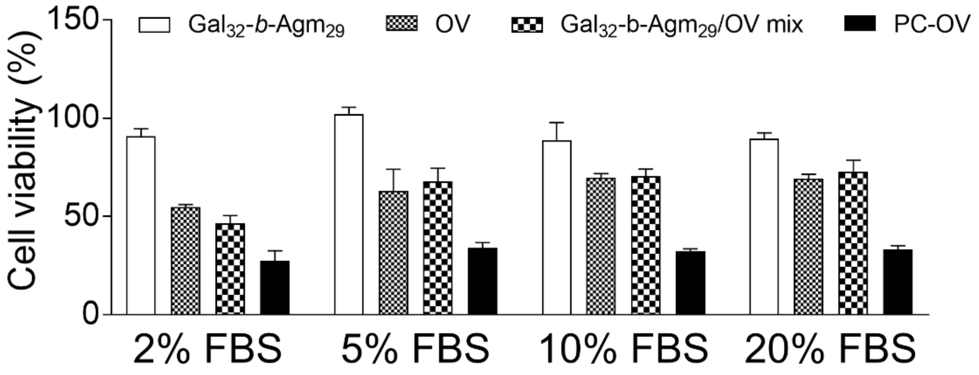

Figure S15. In vitro cytotoxicity on HepG2 cells of naked OV, Gal_{32}-*b*-Agm_{29} alone, Gal_{32}-*b*-Agm_{29}/OV mix and PC-OV measured by MTS cell-viability assay at day 3 post-treatment using FBS increasing % in the culture medium. Control untreated cells were used as reference (100% cell viability).

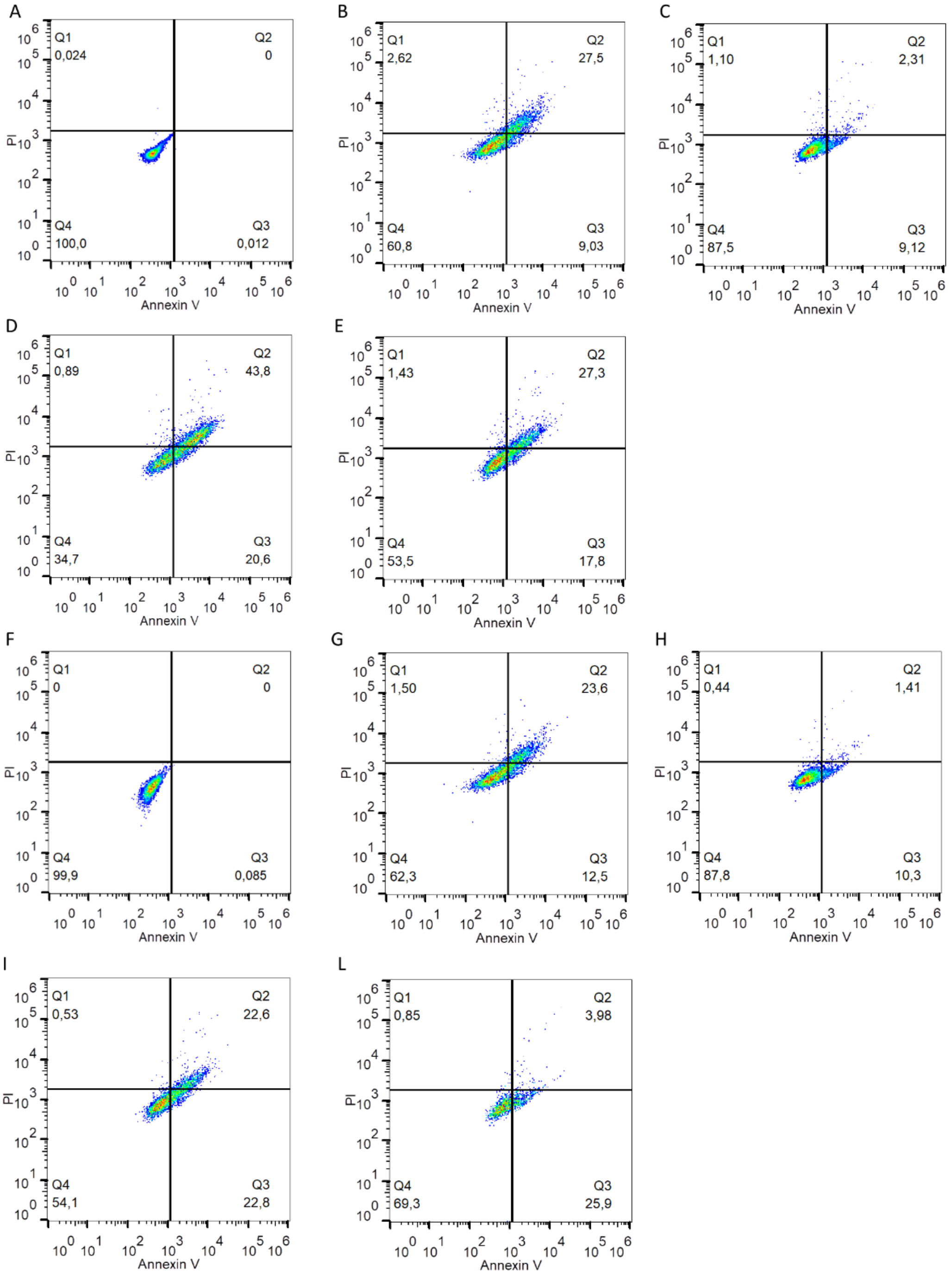

Figure S16. Dot plot with percentage of Annexin V/PI positive HepG2 (A–E) or A549 (F–L) cells, after 48 h treatment. Data shown is representative of at least three independent experiments. (A) and (F): control cells; (B) and (G): virus treated cells; (C) and (H): Gal_{32}-*b*-Agm_{29} treated cells; (D) and (I): PC-OV treated cells; (E) and (L): Gal_{32}-*b*-Agm_{29}/OV mix treated cells.

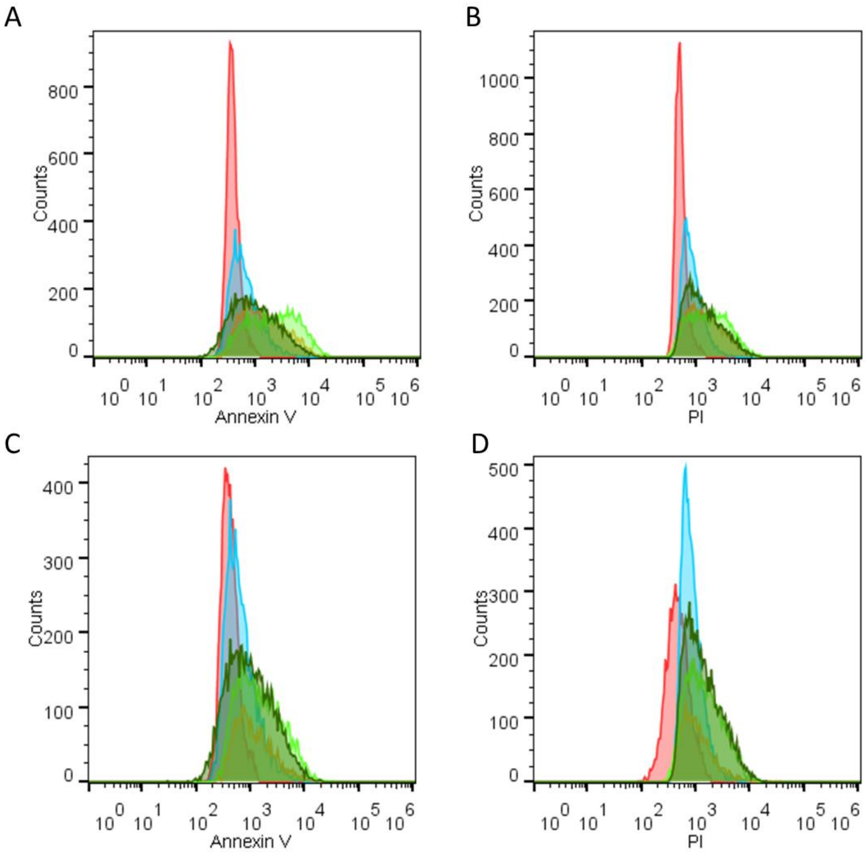

Figure S17. Representative flow cytometry analysis data from Annexin V **(A,C)** / PI **(B,D)** assay performed on HepG2 **(A,B)** and A549 **(C,D)** cells incubated for 48 h with medium only (control cells, red), Gal_{32}-*b*-Agm_{29} polymer (light blue), OV alone (dark green), PC-OV (light green), and Gal_{32}-*b*-Agm_{29}/OV mix (orange).
